# Girth Characterization of (3,29)-Regular Bipartite Graphs and Their Associated LDPC Codes

**DOI:** 10.3390/e28101123

**Published:** 2026-10-14

**Authors:** Hengzhou Xu, Dongshu Chang, Jinlan Guo, Mengmeng Xu, Qian Wang

**Affiliations:** 1 School of Computer, Henan University of Engineering, Zhengzhou 451191, China; hzxu@haue.edu.cn (H.X.); guojinlan1020@163.com (J.G.); mmxu@haue.edu.cn (M.X.); 2 School of Information Science and Engineering, Henan University of Technology, Zhengzhou 450001, China; yuan949826@gmail.com; 3 School of Mathematical Sciences, Shanghai Jiao Tong University, Shanghai 200240, China; 4 School of Mathematics and Statistics, Hubei University of Arts and Science, Xiangyang 441053, China

**Keywords:** bipartite graph, low-density parity-check (LDPC) code, quasi-cyclic (QC), girth, finite field

## Abstract

This paper studies a family of (3,29)-regular bipartite graphs introduced by Tanner for low-density parity-check (LDPC) codes. For a prime p≡1(mod87), the graph has 3p left vertices and 29p right vertices. In the associated LDPC code, the right vertices (there are 29p of them, each of degree 3) serve as the variable nodes, while the left vertices (3p vertices, each of degree 29) serve as the check nodes. Using the Euclidean division algorithm, we completely determine the girth characterization of these graphs. A detailed cycle analysis yields necessary and sufficient conditions for the existence of cycles of lengths 4, 6, 8 and 10, which are translated into polynomial equations over $F_{p}$ involving a primitive 87th root of unity. By computing integer resultants via the Euclidean division algorithm, we identify the prime sets $S_{6}$, $S_{8}$ and $S_{10}$ for which the girth equals 6, 8 or 10, respectively. The sets are constructed recursively so that no prime associated with a shorter cycle is included in a set corresponding to a longer cycle. For all other admissible primes, the girth attains the maximum possible value of 12. Finally, we construct explicit LDPC codes from these graphs and evaluate their error-correcting performance via extrinsic information transfer (EXIT) charts and additive white Gaussian noise (AWGN) simulations. The results indicate competitive performance in the simulated high-rate setting and suggest potential for high-rate applications.

## 1. Introduction

Bipartite graphs play a fundamental role in combinatorial designs, network theory, and coding theory [1,2,3]. In combinatorial designs, they naturally capture incidence relations between points and blocks, leading to structures such as balanced incomplete block designs (BIBDs) and finite geometries [4]. In network theory, bipartite graphs model interactions between two distinct classes of nodes, e.g., users and servers in communication networks or genes and diseases in biological networks [5]. In coding theory, the Tanner graph of a low-density parity-check (LDPC) code is a bipartite graph, and the graph structure directly influences the code’s decoding performance [6,7].

A bipartite graph is called $\left(d_{L},d_{R}\right)$-regular if all vertices in the left part have degree $d_{L}$ and all vertices in the right part have degree $d_{R}$. Such regular structures are particularly attractive for constructing LDPC codes, where the right vertices (degree $d_{R}$) correspond to variable nodes and the left vertices (degree $d_{L}$) correspond to check nodes. Short cycles in the Tanner graph of an LDPC code are known to degrade the performance of iterative decoding algorithms such as the sum-product algorithm (SPA) [8,9,10,11,12,13,14]. The girth, defined as the length of the shortest cycle in the graph, is a fundamental parameter that influences many graph properties [15,16,17]. For instance, large girth improves expansion and connectivity, and in iterative decoding, it mitigates the harmful effects of short cycles and achieves better error-correction performance. Consequently, determining the girth characterization of regular bipartite graph families is a problem of both theoretical interest and practical importance [18,19,20]. Quasi-cyclic LDPC (QC-LDPC) codes have attracted sustained attention in recent years due to their structured parity-check matrices, which enable linear-time encoding with shift registers and highly parallel decoder implementations [21]. These features have made QC-LDPC codes the standard choice in several industrial standards, including 5G New Radio and CCSDS.

A prominent family of such graphs arises from the quasi-cyclic (QC) construction proposed by Tanner [22], which is based on finite fields. For given degrees (J,L), the resulting Tanner graph is a (J,L)-regular bipartite graph with Jp left vertices and Lp right vertices, where *p* is a prime satisfying p≡1(modJL). In this construction, the adjacency is determined by a primitive JLth root of unity in $F_{p}$. A QC-LDPC code is a linear block code whose parity-check matrix is composed of circulant permutation matrices (CPMs); this structure facilitates efficient encoding and decoding in hardware. For the graphs considered here, the parity-check matrix of the associated LDPC code is exactly the incidence matrix of the bipartite graph, where each p×p circulant corresponds to a specific shift. Due to the fully connected circulant structure, the girth of such a graph is always even and is bounded above by 12 [23]; its exact value depends on the chosen prime *p*. Determining which primes yield a given girth is a nontrivial combinatorial problem. This problem has been solved for several (J,L) pairs with J=3 [24,25,26,27,28,29,30,31]. In each case, the majority of admissible primes yield the maximum girth 12, while only a finite set of exceptional primes lead to smaller girths (6, 8 or 10).

The study of this specific (3,29)-regular family is motivated by both practical and theoretical considerations. The associated LDPC code has length 29p, where *p* is the lifting factor, and its design rate is 1−3/29=26/29, which is very high and therefore motivates its study for high-rate applications. The degree profile (3,29) also offers a favorable trade-off between decoding complexity and error-correcting capability. The variable-node degree of 3 keeps the sum-product decoding complexity and latency low, while the check-node degree 29 provides strong parity constraints that help suppress error floors. Moreover, the Tanner graph construction considered here is quasi-cyclic, and this structure enables efficient encoding via shift registers and highly parallel hardware implementation, which is essential for high-throughput systems. From the graph-theoretic viewpoint, the girth of a (3,29)-regular Tanner graph is at most 12 [23]. Short cycles degrade iterative decoding, so graphs attaining girth 12 are especially desirable. Thus, this family provides many high-rate QC-LDPC codes with the maximum possible girth of 12. Finally, the case L=29 fills an important gap in the systematic study of (3,L)-regular Tanner graphs. Previous work has analyzed L=5,7,11,13,17,19,23 [24,25,26,27,28,29,30,31]; L=29 is the next prime value and leads to a substantially higher code rate, while the increased check-node degree, compared with L=23, still remains manageable. This makes the (3,29) family a natural and practically relevant next step. Motivated by these considerations, this paper completes the analysis for the (3,29) case of (3,L)-regular Tanner graphs. We provide a complete characterization of the girth distribution and identify precisely which primes p≡1(mod87) yield each possible girth value. Specifically, we determine three finite exceptional prime sets: $S_{6}$, $S_{8}$, and $S_{10}$. The graph has a girth of 6 if $p\in S_{6}$, a girth of 8 if $p\in S_{8}$, a girth of 10 if $p\in S_{10}$, and a girth of 12 (the maximum possible) for all other admissible primes. Our main contributions are outlined below:

- A complete classification of cycles of lengths 4, 6, 8 and 10 in terms of the underlying prime *p*.
- Translation of cycle existence conditions into polynomial equations over $F_{p}$, and computation of integer resultants (using the Euclidean division algorithm) to determine exactly which primes satisfy those equations.
- Explicit prime sets $S_{6}$, $S_{8}$ and $S_{10}$ with cardinalities $|S_{6}|=5$, $|S_{8}|=196$, $|S_{10}|=4600$. For all other primes p≡1(mod87), the girth is 12.
- Construction of LDPC codes from a representative graph (p=349, girth 8) and AWGN simulation results demonstrating competitive error-correcting performance with fast convergence.

Compared with the previously studied (3,L) cases with L=5,7,11,13,17,19,23, the case L=29 is not merely a quantitative repetition of the same method. The underlying cyclotomic polynomial is $\Phi_{87}\left(x\right)$, whose degree is φ(87)=56, whereas the largest previously encountered case L=23 involves $\Phi_{69}\left(x\right)$ of degree φ(69)=44. More importantly, the enumeration scale grows dramatically: there are 27 valid normalized 6-cycle cases, 1514 normalized 8-cycle cases (757 valid triples for each of the two types), and 21,195 normalized 10-cycle cases. Each case requires an integer resultant computation between a reduced polynomial and $\Phi_{87}\left(t\right)$. In practice, this is carried out by a subresultant pseudo-remainder sequence, during which the coefficients can grow rapidly, leading to coefficient explosion. The final verification is also nontrivial: one must ensure that all candidate primes are correctly extracted, that primes arising only from lower-order roots are excluded, and that no admissible prime is missed. These features make L=29 a substantially more demanding extension of the established framework, and they motivate the more systematic computational and verification strategy.

The remainder of this paper is organized as follows. Section 2 introduces the necessary preliminaries on finite fields and defines the family of (3,29)-regular bipartite graphs. Section 3 presents a systematic classification of cycles of lengths 4, 6, 8, and 10, translating the existence conditions into polynomial equations over $F_{p}$. Using the Euclidean division algorithm, we determine the exact prime sets $S_{6}$, $S_{8}$, and $S_{10}$ for which the girth equals 6, 8, or 10, respectively. Section 4 states the main theorem on the girth characterization, showing that for all other admissible primes, the girth attains the maximum value of 12. Section 5 demonstrates how these graphs yield high-rate LDPC codes and evaluates their error-correcting performance via EXIT charts and numerical simulations. Finally, Section 6 concludes the paper.

## 2. Preliminaries

### 2.1. Finite Fields and Roots of Unity

Let *p* be a prime number such that p≡1(mod87). Then, the finite field $F_{p}$ contains a primitive 87th root of unity, which we denote by λ. In other words, $\lambda^{87}=1$ and $\lambda^{k}\neq 1$ for any positive integer k<87. Because 87=3×29, the elements $\lambda^{29}$ and $\lambda^{3}$ have orders 3 and 29, respectively; hence, they are primitive 3rd and 29th roots of unity. This factorization plays a crucial role in the construction of the Tanner graph, as it naturally separates the contributions of the left and right vertex degrees.

More generally, for any integer *a*, $\lambda^{a}=1$ if and only if 87∣a. The multiplicative group $F_{p}^{*}$ is cyclic of order p−1. The condition p≡1(mod87) is precisely the necessary and sufficient condition for $F_{p}^{*}$ to contain an element of order 87, i.e., a primitive 87th root of unity. Consequently, such a λ exists for every prime *p* in the arithmetic progression 1mod87. By Dirichlet’s theorem on arithmetic progressions (which states that for any coprime positive integers *a* and *d*, there are infinitely many primes of the form a+kd), there are infinitely many such primes. For our purposes, a=1 and d=87.

### 2.2. Definition of the Bipartite Graph Family

For a prime *p* with p≡1(mod87) and a fixed primitive 87th root $\lambda \in F_{p}$, define a bipartite graph G(p) with left part

$$L=\{\left(x,i\right):x\in \left\{0,1,2\right\},\;i\in Z_{p}\}$$

and right part

$$R=\{\left(y,j\right):y\in \left\{0,1,\ldots ,28\right\},\;j\in Z_{p}\}.$$

A left vertex (x,i) is adjacent to a right vertex (y,j) if and only if

(1) $$j-i\equiv \lambda^{29x+3y}\;\left(\mathrm{mod}\;p\right).$$

In graph-theoretic terms, G(p) has a left degree of 29 and a right degree of 3, so it is (29,3)-regular. In coding theory, however, it is customary to list the variable-node degree first and the check-node degree second. Since the right vertices (degree 3) are variable nodes and the left vertices (degree 29) are check nodes, we refer to G(p) as a (3,29)-regular Tanner graph. This coding convention is used consistently throughout the paper, including the title.

Because the map $y\mapsto \lambda^{29x+3y}$ is a bijection from {0,1,…,28} onto the set $\left\{\lambda^{29x},\lambda^{29x+3},\ldots ,\lambda^{29x+84}\right\}$, each left vertex (x,i) has exactly 29 neighbors, namely one for each *y* value. Indeed, as *y* runs over its 29 distinct residues modulo 29, the exponents 29x+3y run over a complete set of residues modulo 87 that are congruent to 29x modulo 3. Hence, the shifts $\lambda^{29x+3y}$ are all distinct, so each left vertex has 29 distinct right neighbors.

Similarly, for a fixed right vertex (y,j), the adjacency condition $j-i\equiv \lambda^{29x+3y}\;\left(\mathrm{mod}\;p\right)$ can be rewritten as $i\equiv j-\lambda^{29x+3y}\;\left(\mathrm{mod}\;p\right)$. For each of the three possible *x* values (0,1,2), the right-hand side determines a unique left vertex index *i*. Thus, (y,j) is adjacent to exactly three left vertices (one for each *x*). Consequently, each right vertex has a degree of 3.

Therefore, G(p) is a (3,29)-regular bipartite graph. The total numbers of vertices are |L|=3p and |R|=29p. For the associated LDPC code, the 29p right vertices (each of degree 3) are taken as the variable nodes, and the 3p left vertices (each of degree 29) are taken as the check nodes. We note that although the construction depends on the choice of the primitive 87th root λ, different choices yield isomorphic graphs. Indeed, if $\lambda^{\prime }$ is another primitive root, then $\lambda^{\prime }=\lambda^{c}$ for some *c* coprime to 87; the automorphism described in the following then gives an isomorphism between the corresponding graphs. Consequently, the girth of G(p) is independent of the choice of λ, and G(p) is well defined up to isomorphism.

### 2.3. Cycle Condition

A closed non-backtracking walk of length 2h in G(p) is an alternating sequence

$$\left(x_{0},i_{0}\right),\left(y_{0},j_{0}\right),\left(x_{1},i_{1}\right),\left(y_{1},j_{1}\right),\ldots ,\left(x_{h-1},i_{h-1}\right),\left(y_{h-1},j_{h-1}\right),\left(x_{0},i_{0}\right)$$

such that consecutive vertices are adjacent and no edge is immediately traversed in the reverse direction. Equivalently, the right indices satisfy $y_{a}\neq y_{a+1}$ and the left indices satisfy $x_{a}\neq x_{a+1}$ cyclically. A simple cycle is a closed non-backtracking walk in which all vertices are distinct except for the starting and ending vertex. In a bipartite graph, every simple cycle has even length.

A cycle of length 2h (called 2h-cycle) in G(p) corresponds to a closed alternating walk of left and right vertices. Because the graph is bipartite, all cycles have even length. Following the standard technique for Tanner graphs [23], a 2h-cycle can be represented by a sequence of right vertices $\left(y_{0},y_{1},\ldots ,y_{h-1}\right)$ with $y_{a}\in \left\{0,\ldots ,28\right\}$ and $y_{a}\neq y_{a+1}$ (indices taken modulo *h*). The left vertices are then uniquely determined by the adjacency rule (1). More precisely, starting from an arbitrary left vertex $\left(x_{0},i_{0}\right)$, the cycle alternates: right vertex $\left(y_{0},j_{0}\right)$ with $j_{0}=i_{0}+\lambda^{29x_{0}+3y_{0}}$, then left vertex $\left(x_{1},i_{1}\right)$ with $i_{1}=j_{0}-\lambda^{29x_{1}+3y_{0}}$, and so on. The condition that the walk closes after 2h steps leads to a constraint on the $y_{a}$’s and the $x_{a}$’s.

The necessary and sufficient condition for the existence of such a cycle is

(2) $$\sum_{a=0}^{h-1}\left(\lambda^{29x_{a}+3y_{a}}-\lambda^{29x_{a+1}+3y_{a}}\right)\equiv 0\;\left(\mathrm{mod}\;p\right),$$

where $x_{a}$ is the unique element of {0,1,2} that makes the adjacency hold, and indices are cyclic with $x_{h}=x_{0}$, $y_{h}=y_{0}$. This equation is obtained by summing the differences $i_{a+1}-i_{a}$ around the cycle; the cancellations of the $i_{a}$’s yield the condition that the sum of the shifts must be zero modulo *p*. We note that due to the translation symmetry of the graph (shifting the indices of left and right vertices) and the invariance of a cycle under cyclic shifts of its vertices, we may assume without loss of generality that the starting right vertex satisfies $y_{0}=0$ and that the initial left vertex can be chosen arbitrarily.

For the graph defined above, the left vertices are indexed by (x,i). The pattern of $x_{a}$ along the cycle is not arbitrary: consecutive left vertices must be distinct, and because the graph is (3,29)-regular, the $x_{a}$ values change in a controlled way.

For cycles of length up to 10 (i.e., h=2,3,4,5), the possible sequences of the $x_{a}$’s are not arbitrary. The $x_{a}$’s form a closed walk of length *h* on the set {0,1,2} with the condition that cyclically adjacent symbols are distinct (since consecutive left vertices in a cycle cannot be the same). Up to rotation, reflection, and the cyclic relabeling x↦x+1 induced by the automorphism $\left(x,i\right)\mapsto \left(x+1,\lambda^{29}i\right)$, $\left(y,j\right)\mapsto \left(y,\lambda^{29}j\right)$, there is exactly one admissible pattern for h=3 and h=5, namely (0,1,2) and (0,1,2,0,1), respectively. For h=4, there are exactly two admissible patterns: (0,1,0,1) and (0,1,0,2). With these patterns fixed, the cycle type is then completely determined by the right-index sequence $\left(y_{0},y_{1},\ldots ,y_{h-1}\right)$, which greatly simplifies the subsequent analysis.

To see that the above classification of *x*-patterns is exhaustive for cycles of length up to 10, consider all closed walks of length *h* on the three symbols {0,1,2} with no cyclically adjacent repeats. For h=3, the only such walk up to the stated symmetries is (0,1,2). For h=4, the possible sequences (up to rotation and reflection) are (0,1,0,2), (0,1,2,1), and (0,1,0,1). The sequence (0,1,2,1) is equivalent to (0,1,0,2) under the cyclic relabeling x↦x+2 together with a cyclic shift. Thus, only two types remain: (0,1,0,1) and (0,1,0,2). For h=5, a direct enumeration yields only one pattern up to symmetry, namely (0,1,2,0,1). Therefore, the patterns used in the following sections are exhaustive for cycles of lengths 8 and 10.

**Remark** **1.** *The cycle condition* (2) *characterizes closed non-backtracking walks, not necessarily simple cycles. In the following, we therefore always exclude shorter cycles first; this guarantees that the walks counted are simple cycles.*

## 3. Cycle Classification up to Length 10

We note that the complexity of the enumeration grows significantly with *L*, and the case L=29 introduces several new mathematical and computational challenges within the Euclidean-division framework. First, the relevant cyclotomic polynomial is $\Phi_{87}\left(x\right)$, whose degree is φ(87)=56. This is higher than in all previously treated cases; for comparison, the largest earlier case L=23 involves $\Phi_{69}\left(x\right)$ of degree φ(69)=44. Second, the number of valid difference combinations increases substantially: there are 27 valid normalized 6-cycle cases, 1514 normalized 8-cycle cases (757 valid triples for each of the two types), and 21,195 normalized 10-cycle cases. This is considerably larger than the corresponding counts for smaller values of *L*, where the analysis involved far fewer cases and lower-degree cyclotomic polynomials. Third, each case requires an integer resultant computation between a reduced polynomial and $\Phi_{87}\left(t\right)$. In practice, this is carried out by a subresultant pseudo-remainder sequence, during which the coefficients can grow rapidly, leading to coefficient explosion. Fourth, the resulting prime sets must be verified carefully: one has to exclude primes that arise only from lower-order factors such as $\Phi_{3}\left(x\right)$ or $\Phi_{29}\left(x\right)$, and one has to confirm that every candidate prime *p* indeed satisfies the original modular equation. To make the computation tractable and the results verifiable, we (i) normalize the first difference to d=1, (ii) exploit cyclic shifts and reversal to reduce equivalent cases, (iii) precompute the power table for $m=\lambda^{3d+29}$, (iv) compute all integer resultants using exact arithmetic over Z, and (v) independently verify each candidate prime by direct modular evaluation.

### 3.1. Normalization by a Girth-Preserving Automorphism

In the cycle analysis below, we will frequently reduce the enumeration to the case where the first difference $d=y_{1}-y_{0}$ is fixed to a nonzero representative, e.g., d=1. This reduction is not merely a convenience; it is justified by a girth-preserving automorphism of the graph family that scales all *y*-differences by an arbitrary nonzero factor modulo 29.

**Lemma** **1** (Root-independence up to isomorphism)**.** *Let p≡1(mod87). Let $\lambda ,\mu \in F_{p}$ be two primitive *87*th roots of unity. Then, the Tanner graphs $G_{\lambda }\left(p\right)$ and $G_{\mu }\left(p\right)$ are isomorphic. Consequently, the girth of G(p) is independent of the choice of the primitive *87*th root.*

**Proof.**  Since both λ and μ are primitive 87th roots of unity, there exists $c\in Z_{87}^{*}$ such that $\mu =\lambda^{c}$. Write

c≡α(mod3),c≡β(mod29),

where α∈{1,2} and $\beta \in Z_{29}^{*}$. Define a map

$$\phi_{c}:\;G_{\lambda }\left(p\right)⟶G_{\mu }\left(p\right)$$

by

$$\phi_{c}\left(x,i\right)=\left(\alpha^{-1}x\;\mathrm{mod}\;3,\;i\right),\;\phi_{c}\left(y,j\right)=\left(\beta^{-1}y\;\mathrm{mod}\;29,\;j\right).$$

The maps $x\mapsto \alpha^{-1}x$ on $Z_{3}$ and $y\mapsto \beta^{-1}y$ on $Z_{29}$ are bijections, so $\phi_{c}$ is a bijection on vertices.

It remains to check that adjacency is preserved. In $G_{\lambda }\left(p\right)$, a left vertex (x,i) is adjacent to a right vertex (y,j) if and only if

$$j-i\equiv \lambda^{29x+3y}\;\left(\mathrm{mod}\;p\right).$$

Under $\phi_{c}$, these vertices map to

$$\left(x^{\prime },i\right)=\left(\alpha^{-1}x\;\mathrm{mod}\;3,\;i\right),\;\left(y^{\prime },j\right)=\left(\beta^{-1}y\;\mathrm{mod}\;29,\;j\right).$$

In the target graph $G_{\mu }\left(p\right)=G_{\lambda^{c}}\left(p\right)$, the adjacency condition for the images is

$$j-i\equiv \mu^{29x^{\prime }+3y^{\prime }}=\lambda^{c(29x^{\prime }+3y^{\prime })}\;\left(\mathrm{mod}\;p\right).$$

We compute the exponent modulo 87:

$$c\left(29x^{\prime }+3y^{\prime }\right)=29\left(cx^{\prime }\right)+3\left(cy^{\prime }\right).$$

Since c≡α(mod3) and $x^{\prime }=\alpha^{-1}x\;\mathrm{mod}\;3$, we have

$$cx^{\prime }\equiv \alpha \alpha^{-1}x\equiv x\;\left(\mathrm{mod}\;3\right),$$

hence $29\left(cx^{\prime }\right)\equiv 29x\;\left(\mathrm{mod}\;87\right)$. Similarly, since c≡β(mod29) and $y^{\prime }=\beta^{-1}y\;\mathrm{mod}\;29$,

$$cy^{\prime }\equiv \beta \beta^{-1}y\equiv y\;\left(\mathrm{mod}\;29\right),$$

hence $3\left(cy^{\prime }\right)\equiv 3y\;\left(\mathrm{mod}\;87\right)$. Therefore

$$c\left(29x^{\prime }+3y^{\prime }\right)\equiv 29x+3y\;\left(\mathrm{mod}\;87\right),$$

and the adjacency condition in $G_{\mu }\left(p\right)$ becomes

$$j-i\equiv \lambda^{29x+3y}\;\left(\mathrm{mod}\;p\right),$$

which is exactly the adjacency condition in $G_{\lambda }\left(p\right)$. Thus, $\phi_{c}$ is an isomorphism. Since any two primitive 87th roots differ by some $c\in Z_{87}^{*}$, all graphs $G_{\lambda }\left(p\right)$ are isomorphic, and the girth is independent of λ. □

**Corollary** **1.** For the purpose of determining whether G(p) contains a cycle of length 2h for h≤5, we may assume without loss of generality that the first right-index difference $d=y_{1}-y_{0}$ is equal to *1*. Indeed, choose β=d(mod29) and any α∈{1,2}, and let $c\in Z_{87}^{*}$ satisfy c≡α(mod3) and c≡β(mod29). By Lemma 1, the graph $G_{\lambda }\left(p\right)$ is isomorphic to $G_{\lambda^{c}}\left(p\right)$, and in the latter, the corresponding difference is

$$\beta^{-1}d\equiv d^{-1}d\equiv 1\;\left(\mathrm{mod}\;29\right).$$

### 3.2. General Method

For each h=2,3,4,5 (corresponding to cycles of length 4,6,8,10), we enumerate all possible sequences $\left(y_{0},\ldots ,y_{h-1}\right)$ that can give a cycle. Using the substitution $u=\lambda^{29}$, $v=\lambda^{3}$, Equation (2) simplifies because each term contains a factor $u^{x_{a}}-u^{x_{a+1}}$. The resulting equation reduces to a polynomial equation in λ (or in $m=\lambda^{3d+29}$ or $M=\lambda^{3d}$). Let $\Phi_{87}\left(t\right)\in Z\left[t\right]$ denote the 87th cyclotomic polynomial. It has degree φ(87)=56 and is irreducible over Q. Since $\lambda \in F_{p}$ is a primitive 87th root of unity, it is a root of the reduction ${\bar{\Phi }}_{87}\left(t\right)\in F_{p}\left[t\right]$ of $\Phi_{87}\left(t\right)$ modulo *p*, i.e.,

$${\bar{\Phi }}_{87}\left(\lambda \right)=0\;\mathrm{in}\;F_{p}.$$

Equivalently, λ satisfies the reduction of $\Phi_{87}$ modulo *p*. We do not speak of a minimal polynomial of λ over Q, because λ is an element of $F_{p}$. The condition p≡1(mod87) guarantees that such a primitive 87th root exists. The polynomial $\Phi_{87}\left(t\right)$ is irreducible over Q; its reduction modulo *p* may factor over $F_{p}$, and when p≡1(mod87), it splits into linear factors over $F_{p}$. The existence of a cycle is equivalent to the existence of a solution to the reduced equation that also satisfies the reduction of $\Phi_{87}$ modulo *p* at λ; i.e., λ is exactly a primitive 87th root—not a lower order root.

For each reduced polynomial P(t), we compute the integer resultant

$$R_{P}=\mathrm{Res}\left(P,\Phi_{r}\right)\in Z,\;r=\left\{\begin{array}{cc} 87, & t=m=\lambda^{3d+29},\\ 29, & t=M=\lambda^{3d}, \end{array}\right.$$

using an exact subresultant pseudo-remainder sequence over Z, and factor it over Z. The candidate primes are precisely the prime divisors of $R_{P}$ satisfying p≡1(mod87) and p∤lc(P).

This method, first used in [24], reduces the problem to checking a finite set of primes for each case. We apply it systematically for all admissible parameter combinations. We now describe in detail the computational method used to determine, for each cycle type, which primes admit a cycle. The method is applied uniformly across all cases in the following subsections.

Step 1: Derivation of the polynomial equation. For a given cycle type with fixed *x*-pattern and right-index differences expressed in terms of d,e,…, substitution into the general cycle condition (2) yields, after simplification, a polynomial equation of the form

P(t)=0,

where *t* is either $m=\lambda^{3d+29}$ (for patterns involving x=0,1,2) or $M=\lambda^{3d}$ (for patterns involving only x=0,1). The exponents in *P* are integer combinations of the remaining differences (e.g., e,f,g) modulo the appropriate order (87 for *m*, 29 for *M*).

Step 2: Reduction via the cyclotomic polynomial. Since $m\in F_{p}$ is a primitive 87th root of unity, it satisfies the reduction modulo *p* of the 87th cyclotomic polynomial:

$${\bar{\Phi }}_{87}\left(m\right)=0\;\mathrm{in}\;F_{p},$$

where the integer polynomial $\Phi_{87}\left(t\right)$ is defined by

$$\Phi_{87}\left(t\right)=\frac{t^{87}-1}{\prod_{r|87,\;r<87}\Phi_{r}\left(t\right)}=\frac{t^{87}-1}{\left(t-1\right)\Phi_{3}\left(t\right)\Phi_{29}\left(t\right)},$$

with

$$\Phi_{3}\left(t\right)=t^{2}+t+1,\;\Phi_{29}\left(t\right)=t^{28}+t^{27}+\cdots +t+1.$$

Equivalently,

$$\Phi_{87}\left(t\right)=\frac{t^{58}+t^{29}+1}{t^{2}+t+1}.$$

The integer polynomial $\Phi_{87}\left(t\right)$ has degree φ(87)=56 and is irreducible over Q. Its reduction ${\bar{\Phi }}_{87}\in F_{p}\left[t\right]$ is the polynomial whose roots in $F_{p}$ are the primitive 87th roots of unity. We emphasize that the expanded coefficients of $\Phi_{87}\left(t\right)$ are not all positive; the expression $t^{56}+t^{55}+\cdots +t+1$ is not the 87th cyclotomic polynomial. All resultant computations in this paper use the integer polynomial $\Phi_{87}\left(t\right)$ as defined above. For $M=\lambda^{3d}$, which is a primitive 29th root, the relevant integer polynomial is $\Phi_{29}\left(t\right)\;=\;t^{28}+t^{27}+\cdots +t+1$, and *M* satisfies its reduction modulo *p*. For each admissible choice of the remaining differences (e.g., e,f for 8-cycles), we form the polynomial $P_{e,f,\ldots }\left(t\right)$ and compute the integer resultant

$$R_{P}=\mathrm{Res}\left(P_{e,f,\ldots },\Phi_{r}\right)\in Z$$

using an exact subresultant pseudo-remainder sequence over Z. The Euclidean division algorithm and the subresultant pseudo-remainder sequence over Z are used only to compute the integer resultant

$$R_{P}=\mathrm{Res}\left(P,\Phi_{r}\right).$$

The candidate primes are extracted from this integer resultant—not from the numerator of a final rational remainder. In particular, we do not use any unproved assertion that a rational Euclidean remainder and the integer resultant yield the same set of primes.

**Remark** **2.** *The correct integer cyclotomic polynomial $\Phi_{87}\left(t\right)$ is used throughout all computations. In particular, the integer resultant is computed with respect to the integer polynomial*

$$\Phi_{87}\left(t\right)=\frac{t^{87}-1}{\left(t-1\right)\Phi_{3}\left(t\right)\Phi_{29}\left(t\right)},$$

*not to the incorrect expression $t^{56}+t^{55}+\cdots +t+1$. The latter is the sum of all 87th roots of unity and has all coefficients equal to one, whereas $\Phi_{87}\left(t\right)$ has coefficients of both signs.*

Step 3: Interpretation of the integer resultant. For each reduced polynomial P(t), we compute the integer resultant

$$R_{P}=\mathrm{Res}\left(P,\Phi_{r}\right)\in Z,\;r=\left\{\begin{array}{cc} 87, & t=m=\lambda^{3d+29},\\ 29, & t=M=\lambda^{3d}, \end{array}\right.$$

and factor it over Z. The candidate primes are precisely the prime divisors of $R_{P}$ that satisfy p≡1(mod87) and p∤lc(P). This integer resultant is the invariant used for prime extraction; no rational-remainder normalization is invoked. For every reduced polynomial, the exponents are given explicitly in the corresponding subsection. The integer resultant $\mathrm{Res}(P,\Phi_{r})$ is computed directly from the explicit polynomial P(t)—not from an implicit exponent vector.

Step 4: Excluding lower-order roots. The factorization of $t^{87}-1$ over Q into cyclotomic polynomials is

$$t^{87}-1=\left(t-1\right)\Phi_{3}\left(t\right)\Phi_{29}\left(t\right)\Phi_{87}\left(t\right),$$

where $\Phi_{3}\left(t\right)=t^{2}+t+1$ and $\Phi_{29}\left(t\right)=t^{28}+\cdots +t+1$. The polynomial $\Phi_{87}$ itself is not the product of these factors; it is the remaining cyclotomic factor. Since we require *m* to be a primitive 87th root, we must exclude solutions that satisfy any lower-order factor. In practice, this is handled by using the full integer cyclotomic polynomial $\Phi_{87}\left(t\right)$ in the resultant

$$\mathrm{Res}(P,\Phi_{87}),$$

so that any prime arising only from a lower-order factor is discarded.

We illustrate the method with a concrete example. For the case (d,e)=(d,2d), there the reduced equation is

$$P\left(m\right)=m^{32}+m+1=0.$$

Using the subresultant pseudo-remainder sequence over Z, we obtain the integer resultant

$$\mathrm{Res}\left(P,\Phi_{87}\right)=\mathrm{Res}\left(m^{32}+m+1,\Phi_{87}\left(m\right)\right)=842006677.$$

Its prime factorization is

$$842006677=29^{2}\cdot 1001197.$$

The factor 29 does not satisfy p≡1(mod87), so it is not admissible. The unique admissible prime factor is

1001197,

which satisfies

1001197≡1(mod87),

and does not divide the leading coefficient of *P*, which is 1. The prime 1001197 admits a primitive 87th root of unity $m\in F_{1001197}$ satisfying $m^{32}+m+1=0$.

We emphasize that the integer resultant and its admissible prime factors are distinct objects: the resultant may contain extraneous prime factors that do not lie in the arithmetic progression 1mod87, and these factors must be discarded. The candidate primes for a given cycle type are precisely the prime divisors of the resultant that satisfy p≡1(mod87) and p∤lc(P).

As a modular verification, the verification script finds an explicit primitive 87th root $m\in F_{1001197}$ and checks that

$$m^{87}=1,\;m^{3}\neq 1,\;m^{29}\neq 1,$$

and

$$m^{32}+m+1=0\;\mathrm{in}\;F_{1001197}.$$

This confirms that $1001197\in S_{6}$, which is in agreement with Table 1. The same resultant-based procedure is applied to every reduced polynomial in the 6-, 8-, and 10-cycle analyses.

**Table 1:** Analysis of (d,e) for 6-cycles. The notation (d,kd) denotes e≡kd(mod29). The reduced equation is obtained by substituting into the cycle condition and converting to powers of *m* using Table 2. All resultant computations use the correct integer cyclotomic polynomial $\Phi_{87}\left(t\right)=\left(t^{87}-1\right)/\left(\left(t-1\right)\Phi_{3}\left(t\right)\Phi_{29}\left(t\right)\right)$. The integer resultant $\mathrm{Res}(P,\Phi_{87})$ may contain extraneous prime factors not satisfying p≡1(mod87); only the admissible prime factors are listed in the last column.

Case	(d,e)	Reduced Equation $m^{k}+m+1=0$	Candidate Primes *p*
1	(d,d)	$m^{2}+m+1$	none
2	(d,2d)	$m^{32}+m+1$	1,001,197
3	(d,3d)	$m^{62}+m+1$	3,768,493
4	(d,4d)	$m^{5}+m+1$	5,233,307
5	(d,5d)	$m^{35}+m+1$	5,233,307
6	(d,6d)	$m^{65}+m+1$	5,233,307
7	(d,7d)	$m^{8}+m+1$	3,768,493
8	(d,8d)	$m^{38}+m+1$	2,293,321
9	(d,9d)	$m^{68}+m+1$	1,001,197
10	(d,10d)	$m^{11}+m+1$	3,768,493
11	(d,11d)	$m^{41}+m+1$	2,293,321
12	(d,12d)	$m^{71}+m+1$	2,293,321
13	(d,13d)	$m^{14}+m+1$	1,001,197
14	(d,14d)	$m^{44}+m+1$	none
15	(d,−d)	invalid	–
16	(d,−2d)	$m^{86}+m+1$	none
17	(d,−3d)	$m^{56}+m+1$	1,001,197
18	(d,−4d)	$m^{26}+m+1$	3,768,493
19	(d,−5d)	$m^{83}+m+1$	5,233,307
20	(d,−6d)	$m^{53}+m+1$	5,233,307
21	(d,−7d)	$m^{23}+m+1$	none
22	(d,−8d)	$m^{80}+m+1$	3,768,493
23	(d,−9d)	$m^{50}+m+1$	2,293,321
24	(d,−10d)	$m^{20}+m+1$	1,001,197
25	(d,−11d)	$m^{77}+m+1$	3,768,493
26	(d,−12d)	$m^{47}+m+1$	2,293,321
27	(d,−13d)	$m^{17}+m+1$	2,293,321
28	(d,−14d)	$m^{74}+m+1$	1,001,197

For every cycle type considered in this paper, the reduced polynomial P(t) is obtained by the following three steps:

1. Substitute the fixed *x*-pattern into the general cycle condition (2);
2. Express every power of λ in terms of the chosen variable *t*, where $t=m=\lambda^{3d+29}$ for patterns involving x=0,1,2, and $t=M=\lambda^{3d}$ for patterns involving only x=0,1;
3. Clear only those denominators that are invertible in $F_{p}$, i.e., powers of λ and of *t*.

No sign changes are introduced in this reduction. In particular, an alternating-sign expression such as $1-M+M\lambda^{3e}-M\lambda^{3(e+f)}$ remains alternating after the reduction, and the resulting polynomial P(t) is tested directly against the relevant cyclotomic polynomial $\Phi_{29}$ or $\Phi_{87}$. The integer resultant $\mathrm{Res}(P,\Phi_{r})$ is then factored over Z, and the candidate primes are its prime divisors satisfying p≡1(mod87) and p∤lc(P).

### 3.3. Cycles of Length 4

For h=2, the only possible type (up to symmetry) is $\left(y_{0},y_{1}\right)=\left(0,d\right)$ with d≢0(mod29). The pattern of *x* is $x_{0}=0$, $x_{1}=1$ (since $x_{a}\equiv a\;\left(\mathrm{mod}\;3\right)$). Substituting into (2) gives

$$\left(1-\lambda^{29}\right)\left(1-\lambda^{3d}\right)\equiv 0\;\left(\mathrm{mod}\;p\right).$$

Because λ is a primitive 87th root, $\lambda^{29}\neq 1$ and $\lambda^{3d}\neq 1$ (as 3d is not a multiple of 87). Hence, the product is never 0 in $F_{p}$. Therefore,

**Proposition** **1.** *The graph G(p) contains no 4-cycles for any prime p≡1(mod87).*

### 3.4. Cycles of Length 6

For h=3, the cycle type is (0,1,2) up to cyclic shift and reversal. Set $d=y_{1}-y_{0}$, $e=y_{2}-y_{1}$ (mod 29). The degeneracy condition d+e≢0(mod29) must hold (otherwise the cycle collapses). The *x* values are 0,1,2, respectively. Equation (2) reduces to

(3) $$1+\lambda^{3d+29}+\lambda^{3(d+e)-29}\equiv 0\;\left(\mathrm{mod}\;p\right).$$

Let $m=\lambda^{3d+29}$. Note that $m^{87}=1$ and m≠1 because 3d+29 is not a multiple of 87 (since 3d≢58(mod87) for d∈{±1,…,±14}). The exponent 3(d+e)−29 can be expressed as a linear combination of 3d+29 and a multiple of 87; indeed,

3(d+e)−29=(3d+29)+3e−58=(3d+29)+3e+29(mod87).

Table 2 serves as a reference for converting between powers of $m=\lambda^{3d+29}$ and powers of λ during the cycle enumeration. For each exponent *a* of *m*, the corresponding exponent *b* of λ is given by b≡a(3d+29)(mod87). This table is used repeatedly in the derivations of the reduced polynomial equations for cycles of lengths 6 and 8, where expressions involving $\lambda^{3d+29}$ need to be expressed in terms of *m*. Using the precomputed table of powers of *m* (see Table 2), Equation (3) becomes $m^{k}+m+1=0$ for some integer *k* determined by *e* modulo 29. All valid normalized (d,e) pairs are enumerated in Table 1. The table contains 28 rows, one of which is invalid, leaving 27 valid normalized 6-cycle cases. By Corollary 1, we fix d=1 throughout the following enumeration. For each case, we compute the integer resultant $\mathrm{Res}(f_{k},\Phi_{87})$ and factor it over Z. The candidate primes are the prime divisors satisfying p≡1(mod87) and $p∤\mathrm{lc}(f_{k})$. The results are summarized in the last column of Table 1. Table 1 enumerates all valid pairs (d,e) for the 6-cycle type (0,1,2). The notation (d,kd) means e≡kd(mod29), where *k* ranges over the integers such that the pair is non-degenerate. For each case, substitution into the general cycle condition yields a reduced equation of the form $m^{k}+m+1=0$. The exponent *k* is determined by reducing the expression 3(d+e)−29 modulo 87 using the reference table above. The last column lists the primes *p* for which the corresponding polynomial equation has a solution in $F_{p}$; these are obtained by applying the Euclidean division method described in Section 3.2.

Collecting distinct primes from all valid cases, we obtain

$$S_{6}=\left\{523,\;3307,\;1,001,197,\;2,293,321,\;3,768,493\right\}.$$

Thus,

**Theorem** **1.** *The graph G(p) has a girth of 6 if and only if $p\in S_{6}$.*

### 3.5. Cycles of Length 8

Two distinct types occur for 8-cycles: (0,1,0,1) and (0,1,0,2). We treat them separately.

#### 3.5.1. Type (0,1,0,1)

Let $d=y_{1}-y_{0}$, $e=y_{2}-y_{1}$, $f=y_{3}-y_{2}$ (mod 29). The nondegeneracy condition d+e+f≢0(mod29) must hold; otherwise, the cycle collapses. We note that the distinctness conditions for the left vertices (the two with x=0 and the two with x=1) and for the right vertices are automatically satisfied for any nonzero d,e,f with d+e+f≢0(mod29). Indeed, a violation would force one of the differences to be zero or would produce a shorter cycle (length 4 or 6), both of which are excluded by our assumptions and by the results of Section 3.3 and Section 3.4. Hence, no additional prime restrictions arise from these conditions. For this type, the *x* pattern is 0,1,0,1 (repeating every two steps). Substituting into the general cycle condition (2) and simplifying (using $\lambda^{29}\neq 1$) yields

(4) $$1-\lambda^{3d}+\lambda^{3(d+e)}-\lambda^{3(d+e+f)}\equiv 0\;\left(\mathrm{mod}\;p\right).$$

Set $M=\lambda^{3d}$ and $N=\lambda^{3}$, so that $M=N^{d}$ and $N=M^{d^{-1}}$, where $d^{-1}$ is the inverse of *d* modulo 29. Since d≢0(mod29), *M* is a primitive 29th root of unity in $F_{p}$. Then, $\lambda^{3(d+e)}=M\cdot N^{e}$, $\lambda^{3(d+e+f)}=M\cdot N^{e+f}$, and Equation (4) becomes

$$1-M+MN^{e}-MN^{e+f}\equiv 0\;\left(\mathrm{mod}\;p\right).$$

Dividing by *M* (which is invertible) gives

$$M^{-1}-1+N^{e}-N^{e+f}\equiv 0\;\left(\mathrm{mod}\;p\right),$$

or equivalently,

$$M^{28}+N^{e}-N^{e+f}-1\equiv 0\;\left(\mathrm{mod}\;p\right),$$

where we used $M^{29}=1$, hence $M^{-1}=M^{28}$.

We now express *N* in terms of *M*. Since $N=M^{d^{-1}}$, we obtain

$$M^{28}+M^{ed^{-1}}-M^{\left(e+f\right)d^{-1}}-1\equiv 0\;\left(\mathrm{mod}\;p\right).$$

This is a polynomial in *M* with integer coefficients. We emphasize that in odd characteristics, one cannot convert this alternating-sign equation into an all-plus polynomial merely by clearing denominators; the signs are essential. The reduced polynomial is therefore

$$P_{e,f}\left(M\right)=M^{28}+M^{ed^{-1}}-M^{\left(e+f\right)d^{-1}}-1.$$

Because *M* is a primitive 29th root of unity, it satisfies the cyclotomic polynomial

$$\Phi_{29}\left(M\right)=\sum_{i=0}^{28}M^{i}=0.$$

For each admissible triple (d,e,f) (i.e., with d+e+f≢0(mod29)), we compute the integer resultant $\mathrm{Res}(P_{e,f},\Phi_{29})$ and factor it over Z. The candidate primes are precisely the prime divisors of this resultant that satisfy p≡1(mod87) and $p∤\mathrm{lc}(P_{e,f})$.

By Corollary 1, we fix d=1 throughout the following enumeration. The enumeration over e,f∈{±1,…,±14} with 1+e+f≢0(mod29) produces 757 valid triples for this type. Table 3 lists the triples (d,e,f) that are excluded from the 8-cycle enumeration. These triples satisfy d+e+f≡0(mod29), which causes the cycle to collapse: the right vertices are not all distinct, or the walk repeats a left vertex prematurely, resulting in a shorter cycle of length 4 or 6. Since such shorter cycles have already been ruled out for the primes under consideration (see Section 3.3 and Section 3.4), excluding these degenerate triples ensures that the enumeration counts only genuine 8-cycles. The remaining $28^{2}-27=757$ valid triples are then analyzed using the Euclidean division method.

For each valid case, we compute the integer resultant $\mathrm{Res}(P_{e,f},\Phi_{29})$. The resulting candidate primes, after removing those already in $S_{6}$, form a subset of the final $S_{8}$.

#### 3.5.2. Type (0,1,0,2)

For this type, the *x* pattern is 0,1,0,2. With the same notation d,e,f, the simplification of (2) leads to

(5) $$1-\lambda^{3d}-\lambda^{3(d+e)-29}+\lambda^{3(d+e+f)-29}\equiv 0\;\left(\mathrm{mod}\;p\right).$$

It is convenient to set $m=\lambda^{3d+29}$, as in the analysis of 6-cycles. Note that $m^{87}=1$ and m≠1 because 3d+29 is not a multiple of 87 (since 3d≢58(mod87)). Then, $\lambda^{3d}=m\cdot \lambda^{-29}$, $\lambda^{3(d+e)-29}=m\cdot \lambda^{3e-58}$, and $\lambda^{3(d+e+f)-29}=m\cdot \lambda^{3(e+f)-58}$. Substituting these into (5), setting d=1, and using $m=\lambda^{32}$, $\lambda =m^{68}$ (since 32·68≡1(mod87)), Equation (5) simplifies directly to

$$1-m^{30}-m^{30e+59}+m^{30(e+f)+59}=0\;\left(\mathrm{mod}\;p\right),$$

where all exponents are reduced modulo 87. Multiplying by −1 and reordering gives the reduced polynomial

$$P_{e,f}\left(m\right)=m^{30}+m^{30e+59}-m^{30(e+f)+59}-1.$$

Equivalently, in the notation $P_{e,f}\left(m\right)=m^{A}+m^{B}-m^{C}-1$, the exponents are explicitly

A=30,B=30e+59(mod87),C=30(e+f)+59(mod87).

The integer resultant $\mathrm{Res}(P_{e,f},\Phi_{87})$ is computed directly from this explicit polynomial. As in Section 3.5.1, the alternating signs are retained; they cannot be removed in odd characteristics. Because *m* is a primitive 87th root of unity, it satisfies $\Phi_{87}\left(m\right)=0$. For each valid triple, we compute the integer resultant $\mathrm{Res}(P_{e,f},\Phi_{87})$, factor it over Z, and retain the prime divisors satisfying p≡1(mod87). As in the 6-cycle analysis, the integer resultant is computed against the full cyclotomic polynomial $\Phi_{87}\left(m\right)$, so that any prime arising only from a lower-order factor is discarded automatically. For each valid triple we compute the integer resultant $\mathrm{Res}(P_{e,f},\Phi_{87})$, factor it over Z, and retain the prime divisors satisfying p≡1(mod87).

Again, the same set of 757 valid triples (d,e,f) (excluding those in Table 3) is examined, so the two 8-cycle types together give

2×757=1514

normalized 8-cycle cases. After processing all cases and discarding primes that already belong to $S_{6}$, we collect the remaining primes into the set $S_{8}$.

In summary, combining the primes obtained from both 8-cycle types and removing those already in $S_{6}$, we obtain the set $S_{8}$ with $|S_{8}|=196$. Consequently, the graph G(p) has a girth of exactly 8 if and only if $p\in S_{8}$ (with $p\notin S_{6}$). The complete list of primes in $S_{8}$ is provided in Appendix A.

### 3.6. Cycles of Length 10

For 10 cycles, the only type (up to cyclic shift and reversal) is (0,1,2,0,1). Let $d\;=\;y_{1}-y_{0}$, $e=y_{2}-y_{1}$, $f=y_{3}-y_{2}$, $g=y_{4}-y_{3}$ (mod 29). The nondegeneracy condition is d+e+f+g≢0(mod29); otherwise, the cycle collapses. The *x* pattern is 0,1,2,0,1. Substituting into the general cycle condition (2) and simplifying (using $\lambda^{29}\neq 1$) yields

(6) $$1+\lambda^{3d+29}+\lambda^{3(d+e)-29}+\lambda^{3(d+e+f)}-\lambda^{3(d+e+f+g)}\equiv 0\;\left(\mathrm{mod}\;p\right).$$

It is convenient to set $m=\lambda^{3d+29}$, as we did for 6-cycles. Then, $\lambda^{3d}=m\lambda^{-29}$, $\lambda^{3(d+e)-29}=m\lambda^{3e-58}$, $\lambda^{3(d+e+f)}=m\lambda^{3(e+f)-29}$ (after adjusting exponents by multiples of 87), and $\lambda^{3(d+e+f+g)}=m\lambda^{3(e+f+g)-29}$. Substituting these into (6), setting d=1, and using $m=\lambda^{32}$, $\lambda =m^{68}$ (since 32·68≡1(mod87)), Equation (6) simplifies directly to

$$1+m+m^{30e+59}+m^{30(e+f)+30}-m^{30(e+f+g)+30}=0\;\left(\mathrm{mod}\;p\right),$$

where all exponents are reduced to modulo 87. Multiplying by −1 and reordering gives the reduced polynomial

$$P_{e,f,g}\left(m\right)=m^{30(e+f+g)+30}-m^{30(e+f)+30}-m^{30e+59}-m-1.$$

Equivalently, in the notation $P_{e,f,g}\left(m\right)=m^{A}-m^{B}-m^{C}-m^{D}-1$, the exponents are explicitly

A=30(e+f+g)+30mod87,B=30(e+f)+30mod87,C=30e+59mod87,D=1.

The integer resultant $\mathrm{Res}(P_{e,f,g},\Phi_{87})$ is computed directly from this explicit polynomial. The signs are determined by the original cycle equation and are retained as they arise; apart from the multiplication by −1 used to obtain the displayed monic form, no additional sign change is introduced by clearing denominators. For each valid quadruple, we compute $\mathrm{Res}(P_{e,f,g},\Phi_{87})$, factor it over Z, and retain the prime divisors satisfying p≡1(mod87). Because *m* is a primitive 87th root of unity, it satisfies $\Phi_{87}\left(m\right)=0$. However, as before, we must ensure that *m* is not a root of lower order factors; for each valid quadruple, we compute the integer resultant $\mathrm{Res}(P,\Phi_{87})$, factor it over Z, and retain the prime divisors satisfying p≡1(mod87).

By Corollary 1, we fix d=1 throughout the following enumeration. The enumeration over e,f,g∈{±1,…,±14} with 1+e+f+g≢0(mod29) yields 21195 normalized 10-cycle cases. For each valid case, we compute the integer resultant $\mathrm{Res}(P_{e,f,g},\Phi_{87})$. The resulting candidate primes, after removing those already in $S_{6}\cup S_{8}$, constitute the set $S_{10}$ with

$$|S_{10}|=4600.$$

The graph G(p) has a girth of exactly 10 if and only if $p\in S_{10}$ (and $p\notin S_{6}\cup S_{8}$). The complete list of primes in $S_{10}$ is provided in Appendix A.

## 4. Main Result on the Girth Characterization

Combining the detailed analyses of Section 3.3, Section 3.4, Section 3.5 and Section 3.6, we obtain a complete description of the girth of the Tanner (3,29)-regular bipartite graphs G(p) for all primes p≡1(mod87).

We emphasize that for each cycle type, the integer resultant $\mathrm{Res}(P,\Phi_{r})$ may contain prime factors outside the arithmetic progression 1mod87. Only those prime factors that satisfy p≡1(mod87) and p∤lc(P) are admissible; the remaining factors are discarded. The sets $S_{6}$, $S_{8}$, and $S_{10}$ collect precisely the admissible primes.

**Theorem** **2** (Girth characterization)**.** *Let p be a prime with p≡1(mod87), and let G(p) be the Tanner (3,29)-regular bipartite graph. Then, the following apply:*

- *If $p\in S_{6}$ (*5* primes), the girth of G(p) is *6*.*
- *If $p\in S_{8}$ (*196* primes), the girth is *8*.*
- *If $p\in S_{10}$ (*4600* primes), the girth is *10*.*
- *If $p\notin S_{6}\cup S_{8}\cup S_{10}$, the girth reaches the maximum value of *12*.*

*The sets $S_{6}$, $S_{8}$, and $S_{10}$ are pairwise disjoint.*

**Proof.**  The first three cases follow from the cycle classification in Section 3.3, Section 3.4, Section 3.5 and Section 3.6. For the last case, let $p\notin S_{6}\cup S_{8}\cup S_{10}$. By the classification, G(p) contains no cycle of length 4, 6, 8, or 10. On the other hand, the parity-check matrix of G(p) is a 3×29 block matrix of p×p circulant permutation matrices, so the girth upper bound 12 for QC-LDPC codes with circulant permutation matrices applies [23]. Hence, girth(G(p))≤12. Since G(p) is bipartite, all cycles have even length, and the only possible length is 12. Thus, girth(G(p))=12.

It remains to note that the three sets are pairwise disjoint. Indeed, by construction, $S_{6}$ consists of the admissible primes admitting a 6-cycle. $S_{8}$ is obtained from the candidate 8-cycle primes after removing all primes in $S_{6}$. $S_{10}$ is obtained from the candidate 10-cycle primes after removing all primes in $S_{6}\cup S_{8}$. Therefore,

$$S_{6}\cap S_{8}=S_{6}\cap S_{10}=S_{8}\cap S_{10}=⌀.$$

□

The analysis for L=29 required handling significantly more cases than previous work on smaller *L* values. For example, the 8-cycle analysis involved 1514 normalized cases (757 valid triples for each of the two types) and the 10-cycle analysis 21,195 valid quadruples compared to much smaller counts for L=5,7,11,13,17,19,23. The degree of the relevant cyclotomic polynomials also increased from $\Phi_{29}$ to $\Phi_{87}$, requiring a more systematic computational approach. This work thus demonstrates the scalability of the Euclidean division method for larger *L*.

The three exceptional sets are explicitly known:
*S*_6_= {523, 3307, 1001197, 2293321, 3768493},*S*_8_= the set of 196 primes listed in Appendix A,*S*_10_= the set of 4600 primes listed in Appendix A.


Several observations can be made from this distribution. First, the number of primes yielding suboptimal girth (6, 8 or 10) is finite, whereas the set of primes giving the maximum girth 12 is infinite. This is consistent with the pattern observed for smaller *L* values: for (3,L)-regular Tanner graphs with L=5,7,11,13,17,19,23, the exceptional sets are also finite and relatively small compared to the infinite set of primes that achieve a girth of 12 [24]. In the present case, L=29, and the total number of exceptional primes is 5+196+4600=4801, which is still finite. Moreover, the three exceptional sets are pairwise disjoint, so each exceptional prime corresponds to exactly one exact girth value among 6, 8, and 10.

Second, among the exceptional primes, those producing girth 6 are the rarest (only 5), followed by those producing girth 8 (196) and then those producing girth 10 (4600). This hierarchy suggests that as the girth increases, the corresponding prime sets become larger, which is intuitive because the algebraic conditions for longer cycles are less restrictive.

Finally, note that the girth cannot exceed 12 for this family [23]. Since $S_{6}\cup S_{8}\cup S_{10}$ is finite, the relative density of admissible primes p≡1(mod87) yielding girth 12 is 1. Therefore, the family {G(p):p≡1(mod87)} provides an infinite source of (3,29)-regular bipartite graphs with an optimal girth of 12.

**Remark** **3.** *The existence of infinitely many primes p≡1(mod87) with a girth of 12 follows from Dirichlet’s theorem on arithmetic progressions and the fact that $S_{6}\cup S_{8}\cup S_{10}$ is a finite set. Hence, the family contains an infinite subfamily of graphs with the maximum possible girth. The existence of a *12*-cycle for $p\notin S_{6}\cup S_{8}\cup S_{10}$ is not assumed separately. It follows from the girth upper bound *12* in [23] together with the exclusion of all shorter even cycle lengths. The applicability of [23] is direct: the Tanner graph G(p) is the factor graph of a QC-LDPC code whose parity-check matrix consists of p×p circulant permutation matrices, which is precisely the setting of [23]. As an additional validation, we independently searched for cycles for several representative primes outside $S_{6}\cup S_{8}\cup S_{10}$ and confirmed the presence of *12* cycles and the absence of cycles of lengths *4*, *6*, *8*, and *10*.*

## 5. LDPC Codes on (3,29)-Regular Bipartite Graphs

The Tanner graph G(p) is exactly the factor graph of a binary LDPC code: the 29p right vertices (each of degree 3) correspond to variable nodes (code bits), and the 3p left vertices (each of degree 29) correspond to check nodes (parity equations). The adjacency matrix of G(p), with rows indexed by left vertices and columns by right vertices, serves as the parity-check matrix H of the code. Thus, H has 3p rows and 29p columns, matching the number of check nodes and variable nodes, respectively. Because each left vertex has a degree of 29 and each right vertex has a degree of 3, the code is (3,29)-regular in the sense that every variable node participates in 3 checks and every check node involves 29 variables. Its length is n=29p. The number of check nodes is 3p, so the dimension is at least n−3p=26p.

There are two obvious independent linear dependencies among the rows of H: for each x∈{0,1,2}, the sum of the *p* rows corresponding to left vertices (x,i) over all $i\in Z_{p}$ is the all-one vector, and the three block sums are identical. Hence,

rank(H)≤3p−2,

and therefore the dimension satisfies

k≥29p−(3p−2)=26p+2.

For the specific instance p=349 used in our simulations, we computed the exact rank by expressing H as a 3×29 polynomial matrix over $F_{2}\left[z\right]/\left(z^{p}-1\right)$ and evaluating its rank at every *p*-th root of unity in the algebraic closure of $F_{2}$. This gives

rank(H)=3p−2=1045,

and hence

k=29p−(3p−2)=9076.

Thus, the (10121,9076) code is constructed. For general *p*, we do not claim that the rank is exactly 3p−2; we only assert the upper bound rank(H)≤3p−2 and the dimension lower bound k≥26p+2.

### 5.1. Exit Chart Analysis

**Figure 1:**
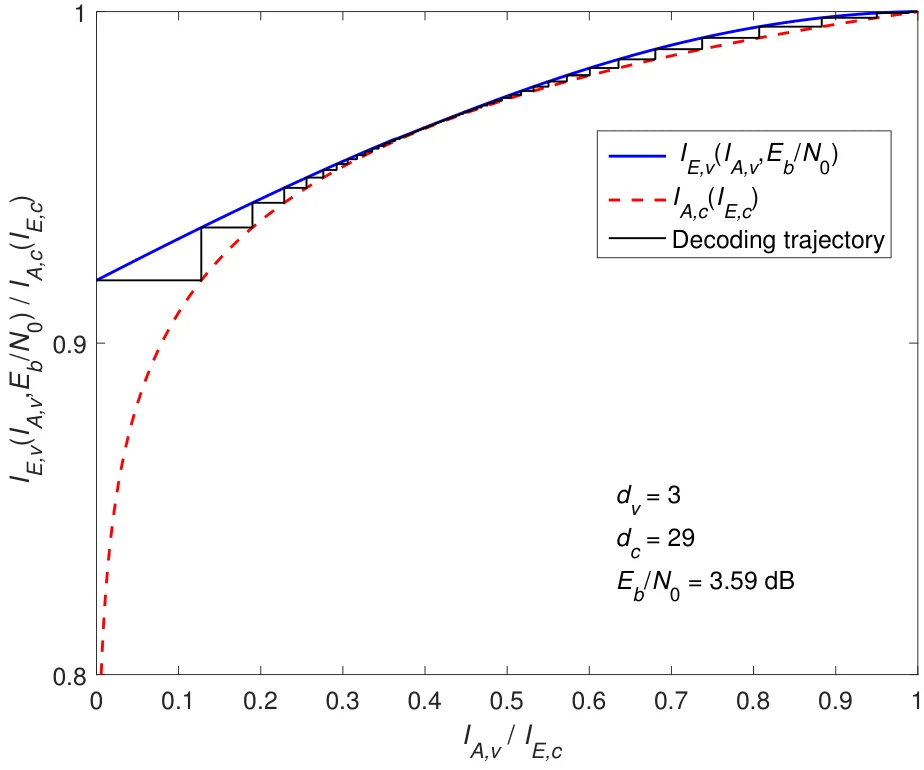
EXIT chart for the (3,29)-regular LDPC code ensemble at threshold $E_{b}/N_{0}=3.59$ dB.

Extrinsic information transfer (EXIT) charts provide a graphical tool to predict the convergence behavior of iterative decoders [32]. The EXIT chart in Figure 1 characterizes the asymptotic (3,29)-regular LDPC ensemble under the Gaussian approximation. It depends only on the degree distribution $\left(d_{v},d_{c}\right)=\left(3,29\right)$, not on the specific prime *p*, the quasi-cyclic structure, the girth, or the short-cycle multiplicity. Therefore, it is used only to estimate the iterative decoding threshold of the ensemble and is not interpreted as evidence that the finite-field construction is superior to other constructions.

We use BPSK modulation with x=1−2c and AWGN y=x+n, $n∼N(0,\sigma^{2})$. The channel LLR is

$$L_{\mathrm{ch}}=\log\frac{p(y|x=+1)}{p(y|x=-1)}=\frac{2y}{\sigma^{2}},$$

whose variance is $\sigma_{\mathrm{ch}}^{2}=4/\sigma^{2}$. For a code of rate *R*, the symbol energy is $E_{s}=1$, and

$$E_{s}/N_{0}=R\;E_{b}/N_{0}.$$

For the ensemble threshold, we use the design rate R=26/29, because the asymptotic ensemble (3,29) has this rate. The finite-length code constructed from G(349) has rate R=9076/10121. Hence,

$$\sigma^{2}=\frac{1}{2E_{s}/N_{0}}=\frac{1}{2R\;E_{b}/N_{0}},$$

and therefore

$$\sigma_{\mathrm{ch}}^{2}=\frac{4}{\sigma^{2}}=8R\;E_{b}/N_{0}.$$

All EXIT and capacity calculations in Section 5.1 and Section 5.2 use this consistent relationship.

Let J(·) be the standard EXIT *J*-function

$$J\left(\sigma \right)=1-\int_{-\infty }^{\infty }\frac{1}{\sqrt{2\pi }\sigma }e^{-{\left(x-\sigma^{2}/2\right)}^{2}/\left(2\sigma^{2}\right)}\log_{2}\left(1+e^{-x}\right)\;dx.$$

The function J(σ) is evaluated by 40-node Gauss–Hermite quadrature on the transformed variable $z=\left(x-\sigma^{2}/2\right)/\left(\sqrt{2}\;\sigma \right)$. The inverse $J^{-1}\left(I\right)$ is obtained by the linear interpolation of a precomputed table ${\left\{\left(\sigma_{i},J\left(\sigma_{i}\right)\right)\right\}}_{i=1}^{1001}$ with $\sigma_{i}$ uniformly spaced on [0,10] with step 0.01. The table is made strictly monotone by enforcing $J\left(\sigma_{i}\right)=\max_{j\leq i}J\left(\sigma_{j}\right)$ and removing duplicate values; every query is clamped to the valid range of the table before interpolation, so no extrapolation occurs.

For a variable node of degree $d_{v}=3$, the VND transfer characteristic is

$$I_{E,v}=J\;\left(\sqrt{\left(d_{v}-1\right)J^{-1}{\left(I_{A,v}\right)}^{2}+\sigma_{\mathrm{ch}}^{2}}\right)=J\;\left(\sqrt{2J^{-1}{\left(I_{A,v}\right)}^{2}+8R\;E_{b}/N_{0}}\right).$$

For a check node of degree $d_{c}=29$, the CND transfer characteristic is

$$I_{E,c}=1-J\;\left(\sqrt{\left(d_{c}-1\right)}\;J^{-1}\left(1-I_{A,c}\right)\right)=1-J\;\left(\sqrt{28}\;J^{-1}\left(1-I_{A,c}\right)\right).$$

The decoding trajectory is generated by iterating

$$I_{A,c}=I_{E,v}\left(I_{A,v}\right),\;I_{A,v}=I_{E,c}\left(I_{A,c}\right),$$

starting from $I_{A,v}=0$. The ensemble threshold is the smallest $E_{b}/N_{0}$ for which the VND curve and the CND curve do not intersect and an open decoding tunnel exists. Numerically, we locate the threshold by bisection on the interval [3.55,3.65] dB with tolerance $10^{-4}$ dB. For each candidate $E_{b}/N_{0}$, the tunnel is declared open iff

$$\Delta \left(I_{A}\right)=I_{E,v}\left(I_{A}\right)-I_{E,c}^{-1}\left(I_{A}\right)>0\;\mathrm{for}\;\mathrm{all}\;I_{A}\in \left(0,1\right),$$

where Δ is evaluated on a sub-grid of 500 uniformly spaced points in (0,1), and $I_{E,c}^{-1}$ is obtained from the same table-based interpolation as $J^{-1}$. The threshold is the smallest $E_{b}/N_{0}$ for which this condition holds. With this setup, we obtain

$$E_{b}/N_{0}^{\mathrm{EXIT}}\approx 3.59\;\mathrm{dB}$$

for the (3,29)-regular ensemble. Replacing linear interpolation by cubic-spline interpolation for $J^{-1}$ and $I_{E,c}^{-1}$ shifts the threshold by less than 0.005 dB, which is below the reported precision. We therefore report the threshold as 3.59 dB. The resulting EXIT chart is shown in Figure 1.

This threshold is an ensemble property and should be clearly distinguished from the performance of the finite p=349 code. The finite-length behavior of the p=349 code, including the effect of its girth 8 and its short cycles, is evaluated by the following numerical simulations.

### 5.2. Simulation Results

The simulations were performed over a binary-input AWGN channel with BPSK modulation. Exploiting the symmetry of the channel and the linearity of the code, the all-zero codeword was transmitted. The sum-product algorithm (SPA) was used for decoding, with maximum iteration limits of 1, 3, 5, 10, 20, 50, and 100.

We constructed a concrete code for the prime p=349, which is the smallest prime in $S_{8}$ and thus yields a graph of girth 8. In $F_{349}$, the element 16 is a primitive 87th root of unity. The corresponding Tanner graph G(349) has a girth of 8 and contains exactly 1,275,246 cycles of length 8. The parity-check matrix H derived from this graph has dimensions 1047 × 10,121, and the resulting code is a (10,121, 9076) QC-LDPC code.

To assess the performance of this code, we compared it with an LDPC code constructed based on the PEG (Progressive Edge Growth) algorithm [33]. The PEG baseline has block length *n* = 10,121 and dimension k=9074, i.e., rate *R* = 9074/10,121, which is approximately matched to the rate of our code, *R* = 9076/10,121 The PEG code uses the same degree profile (3,29) and was simulated under identical BPSK/AWGN conditions with the same SPA decoder and iteration limits.

For the binary-input AWGN channel, the capacity C(σ) is computed numerically by

$$C\left(\sigma \right)=1-\int_{-\infty }^{\infty }\frac{1}{\sqrt{2\pi }\sigma }e^{-{\left(y-1\right)}^{2}/\left(2\sigma^{2}\right)}\log_{2}\;\left(1+e^{-2y/\sigma^{2}}\right)\;dy,$$

where $\sigma^{2}=1/\left(2RE_{b}/N_{0}\right)$. Using the exact code rate *R* = 9076/10,121, the binary-input Shannon limit is the smallest $E_{b}/N_{0}$ such that C(σ)=R; numerically, it is

$$E_{b}/N_{0}^{\mathrm{cap}}\approx 3.15\;\mathrm{dB}.$$

The ensemble EXIT threshold is

$$E_{b}/N_{0}^{\mathrm{EXIT}}\approx 3.59\;\mathrm{dB}.$$

Hence, the ensemble EXIT threshold gap to the capacity limit is

$$\Delta_{\mathrm{EXIT}-\mathrm{cap}}=3.59-3.15\approx 0.44\;\mathrm{dB}.$$

For the finite-length p=349 code, the simulated BER reaches $10^{-6}$ at approximately $E_{b}/N_{0}\approx 4.1$ dB. Therefore,

$$\Delta_{\mathrm{sim}-\mathrm{cap}}\approx 4.1-3.15=0.95\;\mathrm{dB},$$

while its gap to the ensemble EXIT threshold is

$$\Delta_{\mathrm{sim}-\mathrm{EXIT}}\approx 4.1-3.59\approx 0.51\;\mathrm{dB}.$$

**Figure 2:**
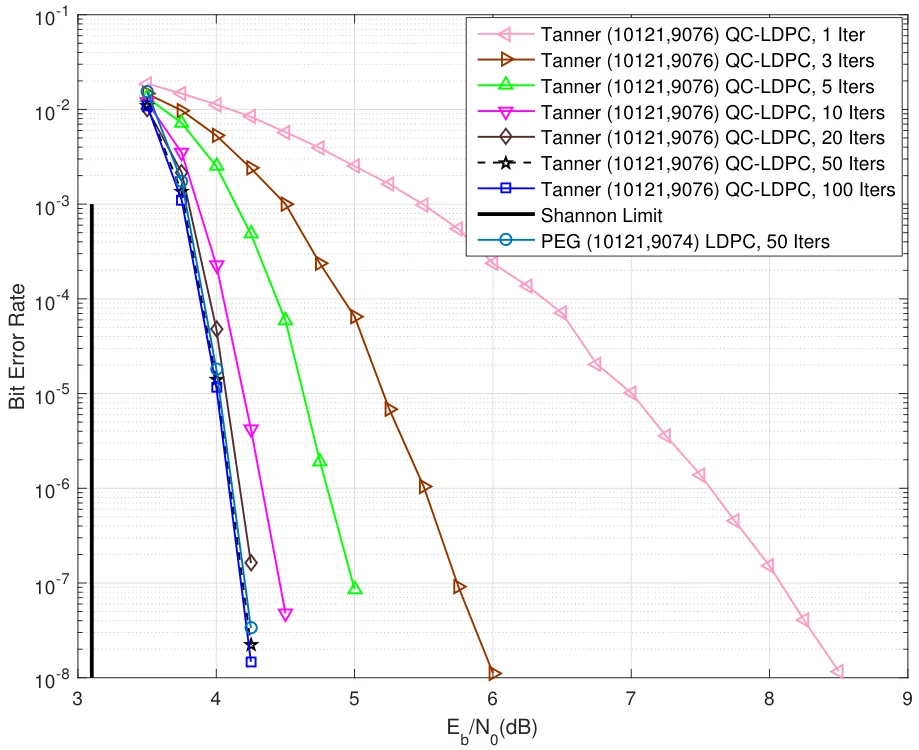
BER performance of the (10,121, 9076) code (p=349, girth 8) and the comparable PEG (10,121, 9074) code under SPA with different iteration limits.

Figure 2 shows the bit error rate (BER) curves of the p=349 code and the PEG baseline. The p=349 code exhibits performance comparable to the PEG baseline at matched rate and length with fast convergence. These results are intended as an illustrative simulation of one code instance—not as a claim of superiority over all existing constructions. At every SNR point, we use a fixed-frame-count scheme: we simulate a fixed number of frames $N_{f}$ and record the number of bit errors $N_{\mathrm{be}}$ and the number of frame errors $N_{\mathrm{fe}}$. The bit error rate and frame error rate are computed directly from these counts as

$$\mathrm{BER}=\frac{N_{\mathrm{be}}}{N_{f}n},\;\mathrm{FER}=\frac{N_{\mathrm{fe}}}{N_{f}},$$

where n=10121 is the block length. These statistics are reported in Table 4 for the 50-iteration case and in Table 5 for the 100-iteration case.

The lowest reliably simulated BER is $1.4\times 10^{-8}$ at $E_{b}/N_{0}=4.25$ dB with 100 iterations. The corresponding counts are $N_{f}=10^{7}$, $N_{\mathrm{be}}=1427$, and $N_{\mathrm{fe}}=131$, giving $\mathrm{BER}\;=\;1.4\;\times \;10^{-8}$ and $\mathrm{FER}=1.3\times 10^{-5}$; see Table 5. The lowest BER in Table 4 (50 iterations) is $2.2\times 10^{-8}$ at the same SNR point of 4.25 dB; the two values therefore refer to different iteration settings rather than to different SNR points. It can be seen from Figure 2 that no error floor was observed down to $\mathrm{BER}=1.4\times 10^{-8}$. Figure 2 additionally shows the 1-, 3-, 5-, 10-, 20-, 50-, and 100-iteration curves. Thus, the results shown only in Figure 2 should be read as iteration-limit comparisons, whereas the quantitative reference for the manuscript is Table 4 and Table 5.

## 6. Conclusions

We have determined the exact girth characterization of a family of (3,29)-regular bipartite graphs constructed by Tanner’s method. The analysis uses a combination of combinatorial cycle enumeration, polynomial equation formulation, and the Euclidean division algorithm. The result is that among all primes p≡1(mod87), only 5 give girth 6, 196 give girth 8, 4600 give girth 10, and the rest give the maximum possible girth 12. The corresponding prime sets $S_{6}$, $S_{8}$, and $S_{10}$ are pairwise disjoint. With the 29p right vertices acting as variable nodes and the 3p left vertices as check nodes, these graphs, when interpreted as Tanner graphs of LDPC codes, yield high-rate codes whose AWGN performance is competitive with a rate-matched PEG baseline in the presented simulation. The EXIT chart predicts an ensemble threshold of about 3.59 dB, and no error floor was observed down to $\mathrm{BER}=1.4\times 10^{-8}$. The work extends the known results for (3,L)-regular graphs to L=29, and it provides a complete characterization that can be used for code design in high-rate applications.

## Figures and Tables

**Table 2:** $m^{a}$ represented by the powers of λ.

$m^{a}$	$\lambda^{b}$	$m^{a}$	$\lambda^{b}$	$m^{a}$	$\lambda^{b}$	$m^{a}$	$\lambda^{b}$	$m^{a}$	$\lambda^{b}$
*m*	$\lambda^{3d+29}$	$m^{2}$	$\lambda^{6d+58}$	$m^{3}$	$\lambda^{9d}$	$m^{4}$	$\lambda^{12d+29}$	$m^{5}$	$\lambda^{15d+58}$
$m^{6}$	$\lambda^{18d}$	$m^{7}$	$\lambda^{21d+29}$	$m^{8}$	$\lambda^{24d+58}$	$m^{9}$	$\lambda^{27d}$	$m^{10}$	$\lambda^{30d+29}$
$m^{11}$	$\lambda^{33d+58}$	$m^{12}$	$\lambda^{36d}$	$m^{13}$	$\lambda^{39d+29}$	$m^{14}$	$\lambda^{42d+58}$	$m^{15}$	$\lambda^{45d}$
$m^{16}$	$\lambda^{48d+29}$	$m^{17}$	$\lambda^{51d+58}$	$m^{18}$	$\lambda^{54d}$	$m^{19}$	$\lambda^{57d+29}$	$m^{20}$	$\lambda^{60d+58}$
$m^{21}$	$\lambda^{63d}$	$m^{22}$	$\lambda^{66d+29}$	$m^{23}$	$\lambda^{69d+58}$	$m^{24}$	$\lambda^{72d}$	$m^{25}$	$\lambda^{75d+29}$
$m^{26}$	$\lambda^{78d+58}$	$m^{27}$	$\lambda^{81d}$	$m^{28}$	$\lambda^{84d+29}$	$m^{29}$	$\lambda^{58}$	$m^{30}$	$\lambda^{3d}$
$m^{31}$	$\lambda^{6d+29}$	$m^{32}$	$\lambda^{9d+58}$	$m^{33}$	$\lambda^{12d}$	$m^{34}$	$\lambda^{15d+29}$	$m^{35}$	$\lambda^{18d+58}$
$m^{36}$	$\lambda^{21d}$	$m^{37}$	$\lambda^{24d+29}$	$m^{38}$	$\lambda^{27d+58}$	$m^{39}$	$\lambda^{30d}$	$m^{40}$	$\lambda^{33d+29}$
$m^{41}$	$\lambda^{36d+58}$	$m^{42}$	$\lambda^{39d}$	$m^{43}$	$\lambda^{42d+29}$	$m^{44}$	$\lambda^{45d+58}$	$m^{45}$	$\lambda^{48d}$
$m^{46}$	$\lambda^{51d+29}$	$m^{47}$	$\lambda^{54d+58}$	$m^{48}$	$\lambda^{57d}$	$m^{49}$	$\lambda^{60d+29}$	$m^{50}$	$\lambda^{63d+58}$
$m^{51}$	$\lambda^{66d}$	$m^{52}$	$\lambda^{69d+29}$	$m^{53}$	$\lambda^{72d+58}$	$m^{54}$	$\lambda^{75d}$	$m^{55}$	$\lambda^{78d+29}$
$m^{56}$	$\lambda^{81d+58}$	$m^{57}$	$\lambda^{84d}$	$m^{58}$	$\lambda^{29}$	$m^{59}$	$\lambda^{3d+58}$	$m^{60}$	$\lambda^{6d}$
$m^{61}$	$\lambda^{9d+29}$	$m^{62}$	$\lambda^{12d+58}$	$m^{63}$	$\lambda^{15d}$	$m^{64}$	$\lambda^{18d+29}$	$m^{65}$	$\lambda^{21d+58}$
$m^{66}$	$\lambda^{24d}$	$m^{67}$	$\lambda^{27d+29}$	$m^{68}$	$\lambda^{30d+58}$	$m^{69}$	$\lambda^{33d}$	$m^{70}$	$\lambda^{36d+29}$
$m^{71}$	$\lambda^{39d+58}$	$m^{72}$	$\lambda^{42d}$	$m^{73}$	$\lambda^{45d+29}$	$m^{74}$	$\lambda^{48d+58}$	$m^{75}$	$\lambda^{51d}$
$m^{76}$	$\lambda^{54d+29}$	$m^{77}$	$\lambda^{57d+58}$	$m^{78}$	$\lambda^{60d}$	$m^{79}$	$\lambda^{63d+29}$	$m^{80}$	$\lambda^{66d+58}$
$m^{81}$	$\lambda^{69d}$	$m^{82}$	$\lambda^{72d+29}$	$m^{83}$	$\lambda^{75d+58}$	$m^{84}$	$\lambda^{78d}$	$m^{85}$	$\lambda^{81d+29}$
$m^{86}$	$\lambda^{84d+58}$	$m^{87}$	$\lambda^{0}\left(=1\right)$						

**Table 3:** Invalid cases (d,e,f) for 8-cycle types (0,1,0,1) and (0,1,0,2). These triples are excluded because they cause the cycle to degenerate into a shorter cycle (length 4 or 6).

(d,e,f) with d+e+f≡0(mod29)				
(d,−14d,13d)	(d,−13d,12d)	(d,−12d,11d)	(d,−11d,10d)	(d,−10d,9d)
(d,−9d,8d)	(d,−8d,7d)	(d,−7d,6d)	(d,−6d,5d)	(d,−5d,4d)
(d,−4d,3d)	(d,−3d,2d)	(d,−2d,d)	(d,d,−2d)	(d,2d,−3d)
(d,3d,−4d)	(d,4d,−5d)	(d,5d,−6d)	(d,6d,−7d)	(d,7d,−8d)
(d,8d,−9d)	(d,9d,−10d)	(d,10d,−11d)	(d,11d,−12d)	(d,12d,−13d)
(d,13d,−14d)	(d,14d,14d)			

Note: The above list includes all triples where d+e+f≡0(mod29).

**Table 4:** Bit/frame error counts for the proposed (10,121, 9076) QC-LDPC code and the comparable PEG (10,121, 9074) code under SPA decoding with 50 iterations over the AWGN channel. $N_{f}$ denotes the number of simulated frames, $N_{\mathrm{be}}$ the number of bit errors and $N_{\mathrm{fe}}$ the number of frame errors.

$E_{b}/N_{0}$	$N_{f}$	Proposed Code				PEG Code			
(dB)		$N_{\mathrm{be}}$	BER	$N_{\mathrm{fe}}$	FER	$N_{\mathrm{be}}$	BER	$N_{\mathrm{fe}}$	FER
3.5	100	11,115	0.0110	84	0.84	15,687	0.0155	88	0.88
3.75	$10^{3}$	13,659	$1.4\times 10^{-3}$	137	0.137	17,590	0.001738	157	0.157
4	$10^{5}$	14,107	$1.4\times 10^{-5}$	182	$1.8\times 10^{-3}$	18,392	$1.8\times 10^{-5}$	203	$2.0\times 10^{-3}$
4.25	$10^{7}$	2229	$2.2\times 10^{-8}$	138	$1.4\times 10^{-5}$	3401	$3.4\times 10^{-8}$	224	$2.2\times 10^{-5}$

**Table 5:** Counts for the proposed (10,121,9076) QC-LDPC code at $E_{b}/N_{0}=4.25$ dB with 100 iterations. $N_{f}$, $N_{\mathrm{be}}$, and $N_{\mathrm{fe}}$ denote the number of simulated frames, bit errors, and frame errors, respectively.

$E_{b}/N_{0}$ (dB)	$N_{f}$	$N_{\mathrm{be}}$	BER	$N_{\mathrm{fe}}$	FER
4.25	$10^{7}$	1427	$1.4\times 10^{-8}$	131	$1.3\times 10^{-5}$

## Data Availability

The data and computational materials that support the findings of this research are available from the corresponding author upon reasonable request.

## Abbreviations

The following abbreviations are used in this manuscript:

LDPC	Low-density parity-check
QC	Quasi-cyclic
BIBD	Balanced incomplete block design
CPM	Circulant permutation matrix
QC-LDPC	Quasi-cyclic low-density parity-check
SPA	Sum-product algorithm
$F_{p}$	Finite field of order *p*
$F_{p}^{*}$	Multiplicative group of order p−1
G(p)	A (3, 29)-regular bipartite graph
p ≡ 1 (modJL)	Condition for existence of primitive JL-th root (for our parameters, JL = 87)
2h-cycle	A cycle of length 2h
$\Phi_{87}\left(x\right)$	87th cyclotomic polynomial
GCD	Greatest common divisor
$S_{6}$	The prime set corresponding to girth 6
$S_{8}$	The prime set corresponding to girth 8
$S_{10}$	The prime set corresponding to girth 10
H	Parity-check matrix
EXIT	Extrinsic information transfer
VND	Variable-node decoder
CND	Check-node decoder
AWGN	Additive white Gaussian noise
BPSK	Binary phase shift keying
SNR	Signal-to-noise ratio
BER	Bit error rate
FER	Frame error rate

## Appendix A. Complete Sets *S*_6_, *S*_8_, and *S*_10_

For reproducibility, this appendix lists the complete exceptional prime sets.

### Appendix A.1. The Set S*_6_*

The set $S_{6}$ contains five primes:

$$S_{6}=\left\{523,\;3307,\;1001197,\;2293321,\;3768493\right\}.$$

### Appendix A.2. The Set S*_8_*

The set $S_{8}$ contains 196 primes:

$S_{8}$ = {349, 1567, 1741, 2089, 2437, 4003, 4177, 5569, 5743, 6091, 6961, 7309, 8353, 8527, 9049, 10093, 10267, 10789, 11311, 11833, 12703, 13399, 13921, 15313, 15661, 16879, 17923, 18097, 19141, 19489, 20011, 20533, 20707, 21577, 21751, 22273, 23143, 29581, 30103, 33409, 33931, 39499, 49069, 49939, 52027, 53593, 55333, 55681, 60901, 62119, 62467, 62989, 68209, 82129, 86131, 87523, 90481, 102139, 105619, 122149, 124759, 137983, 143551, 151903, 155209, 155557, 156601, 215587, 218371, 219763, 230203, 238729, 247603, 251431, 261697, 279619, 294757, 310243, 318247, 339649, 353047, 404029, 428041, 462493, 484417, 503383, 532093, 600823, 639451, 713227, 728017, 749941, 763339, 1019119, 1102117, 1123693, 1136917, 2088349, 2237293, 2420689, 2476717, 2648629, 3033169, 3358549, 3480871, 3866977, 4240381, 4540009, 5034691, 5075929, 6314809, 6468277, 7006981, 7079539, 9447679, 10355263, 15607453, 17679967, 19645471, 19913779, 22118533, 23641903, 26816533, 27951013, 33474121, 37464463, 46163941, 47410651, 47654251, 48001903, 50492539, 50976781, 53449321, 54711169, 69748249, 73938343, 82016989, 100196857, 107055589, 108050869, 113544919, 121171861, 141291307, 202987879, 243140467, 253008181, 256078237, 271222849, 301559053, 302581999, 307988701, 309013213, 309845803, 350448529, 356822149, 431836681, 469462267, 540468883, 569037247, 610178329, 692543491, 768886861, 785849773, 803303191, 836218597, 875865019, 886644841, 938022691, 1453274101, 1456667101, 1496434453, 1602314149, 1621428049, 1772077597, 2112330769, 2125722331, 2499733069, 2521668379, 3126401899, 3420134779, 3478057987, 5004272191, 5428275913, 5816527681, 6366991819, 8061328477, 11528676589, 12847245283, 21779620717, 24155016517, 1016453246203, 3823321050217, 4404459169021, 5271704781907, 9687019108141, 13295270923219}.

### Appendix A.3. The Set S*_10_*

The set $S_{10}$ contains 4600 primes:

$S_{10}$ = {8179, 9397, 12007, 15139, 16183, 17053, 17401, 17749, 18793, 20359, 22447, 22621, 24709, 25057, 25579, 26449, 28537, 28711, 29059, 31147, 31321, 32191, 32713, 32887, 33757, 35149, 35323, 35671, 36541, 38281, 38629, 38803, 38977, 39847, 40543, 41413, 41761, 42283, 42457, 42979, 44371, 44893, 45589, 45763, 46633, 46807, 49417, 50287, 50461, 51157, 51679, 51853, 52201, 53419, 56377, 57073, 57943, 59509, 60727, 63337, 64033, 64381, 65599, 66643, 67339, 69427, 70123, 70297, 71167, 71341, 72211, 72559, 72733, 72907, 73951, 74821, 75169, 76039, 76213, 76387, 76561, 77431, 78301, 78649, 78823, 79693, 79867, 80737, 80911, 82651, 83869, 84391, 84913, 85087, 87697, 88741, 89611, 89959, 91873, 92221, 92569, 93787, 94309, 94483, 95527, 95701, 96223, 97441, 97789, 99181, 99529, 99877, 100747, 103183, 103357, 104053, 105097, 105967, 106663, 107881, 108751, 109621, 110491, 111187, 112927, 113623, 113797, 114319, 114493, 115363, 116929, 117973, 118147, 118669, 118843, 119191, 121453, 122323, 122497, 125803, 126499, 127717, 128239, 128413, 128761, 129457, 129631, 131023, 131371, 131893, 132241, 132589, 132763, 133633, 133981, 134503, 134677, 134851, 135721, 136069, 138157, 138679, 139201, 140071, 140419, 141637, 141811, 142159, 144073, 144247, 145987, 146161, 146857, 147031, 147727, 148249, 149119, 151381, 152599, 152947, 153469, 153817, 153991, 154339, 155383, 155731, 156253, 158341, 158863, 160603, 162343, 162517, 162691, 163561, 163909, 164431, 164953, 166693, 166867, 167911, 168433, 169129, 170347, 171043, 173827, 175393, 176089, 176611, 177481, 178873, 179743, 182179, 182353, 182701, 183397, 183571, 184441, 185833, 186007, 186877, 187573, 187921, 188443, 188791, 190357, 191227, 191749, 192097, 192271, 194707, 195229, 195751, 198013, 199753, 200797, 201493, 201667, 203233, 204973, 206191, 206887, 207061, 207409, 207931, 208279, 208627, 209497, 210019, 210193, 211933, 212281, 213847, 214021, 214891, 215239, 218719, 219937, 220807, 221677, 222199, 223243, 223939, 224113, 224461, 225157, 226027, 226201, 226549, 227419, 227593, 228637, 229507, 229681, 231943, 232117, 232987, 233683, 234727, 236293, 237163, 237859, 238207, 239251, 241687, 241861, 242731, 244297, 245863, 248299, 249517, 251257, 252823, 254041, 254389, 254911, 255259, 256129, 259783, 262567, 263089, 263437, 263611, 264133, 264829, 265003, 266047, 267439, 267613, 267961, 269179, 269527, 269701, 270223, 272659, 275269, 276487, 277183, 277531, 278227, 279967, 280837, 282577, 283099, 283447, 284839, 285709, 286753, 286927, 287449, 288493, 291103, 292147, 297019, 297889, 298237, 298411, 298759, 299977, 300673, 302587, 303283, 304153, 304501, 304849, 305023, 305719, 307633, 312331, 313549, 313897, 315811, 316507, 316681, 317029, 319117, 321553, 322249, 322771, 323467, 326773, 326947, 327469, 328687, 329209, 329557, 334429, 334603, 334777, 335473, 338431, 339127, 341041, 342607, 346261, 347131, 348001, 349567, 350437, 351133, 351829, 353917, 354439, 356701, 358441, 359311, 360007, 360181, 361747, 362443, 364183, 366097, 366967, 368359, 368881, 369751, 372013, 372709, 373231, 373753, 377581, 378277, 381061, 381757, 384193, 384889, 386977, 388369, 394633, 395677, 396199, 396547, 397939, 398113, 398287, 399853, 401593, 403159, 403681, 405247, 409597, 411337, 412033, 414643, 415861, 418993, 425779, 426301, 426997, 430999, 431521, 431869, 432391, 433087, 433261, 434479, 434827, 436741, 437959, 441787, 445789, 447703, 447877, 449269, 452227, 452923, 454141, 455011, 455881, 457099, 457621, 458317, 462667, 462841, 463363, 463537, 465799, 466321, 467017, 469801, 472063, 479023, 480937, 481807, 484243, 486679, 487897, 488419, 490159, 491377, 492421, 499033, 500947, 501121, 502861, 505123, 511387, 512779, 514519, 515041, 515563, 515737, 517999, 522523, 530353, 531571, 532267, 534529, 536443, 538357, 540271, 541837, 542533, 544273, 547753, 549319, 550189, 551059, 551233, 551407, 551581, 552103, 554017, 562021, 563413, 566719, 567937, 572461, 574201, 577333, 580291, 581857, 583249, 585163, 585337, 586903, 591079, 592993, 593689, 593863, 595081, 599779, 603607, 603781, 605347, 607261, 610741, 611263, 612133, 612481, 613699, 614569, 614743, 618049, 621007, 624139, 624487, 630229, 630577, 631273, 632839, 641713, 643453, 646411, 649717, 650413, 655111, 655807, 658591, 659983, 660157, 661897, 662941, 665029, 666073, 666769, 667987, 669379, 669901, 671467, 673207, 678949, 679123, 680341, 682951, 688867, 689041, 694087, 694957, 696523, 698263, 698437, 708703, 710443, 710791, 717229, 723319, 727843, 728191, 728713, 733237, 739153, 742111, 744547, 745243, 745939, 747157, 765949, 766471, 769429, 773953, 774997, 777433, 777781, 779173, 779347, 784219, 788569, 792049, 794659, 795877, 798139, 800053, 800923, 805099, 807187, 809101, 812233, 815713, 817279, 821281, 821803, 828067, 829111, 830329, 839029, 839899, 853819, 855733, 860779, 866869, 867913, 872437, 872611, 874351, 874873, 876787, 882529, 882703, 883921, 884617, 890707, 893317, 893839, 896101, 904627, 904801, 906541, 910369, 915067, 918199, 919591, 921331, 924463, 924811, 926203, 926899, 930379, 931597, 940993, 944473, 951259, 951781, 952129, 959263, 961003, 966919, 968659, 969877, 974749, 979273, 982057, 982231, 986581, 989887, 993541, 994933, 1000849, 1012159, 1012507, 1013029, 1020337, 1022773, 1023643, 1026253, 1030429, 1031299, 1037041, 1039477, 1053571, 1054441, 1057051, 1058791, 1059313, 1064533, 1075843, 1083151, 1084891, 1091329, 1093591, 1097419, 1100899, 1111687, 1114471, 1119343, 1121083, 1121257, 1123867, 1124041, 1133959, 1134481, 1141963, 1142311, 1144399, 1144573, 1145269, 1147009, 1147183, 1149619, 1150141, 1150837, 1158841, 1160581, 1163713, 1167889, 1168411, 1173283, 1173979, 1176937, 1185463, 1191727, 1193989, 1199557, 1200949, 1205473, 1207387, 1213651, 1224439, 1230181, 1230529, 1232269, 1241491, 1242361, 1243579, 1244797, 1247581, 1248103, 1248799, 1251409, 1258717, 1266373, 1273681, 1278379, 1286209, 1291603, 1301173, 1304131, 1308829, 1325011, 1333537, 1339627, 1340323, 1340497, 1341019, 1349371, 1357549, 1362247, 1366597, 1368163, 1369033, 1378603, 1380517, 1390783, 1395481, 1398091, 1400701, 1401571, 1403833, 1406443, 1412011, 1415143, 1422103, 1424191, 1425583, 1436023, 1439329, 1441591, 1443331, 1445419, 1454119, 1458817, 1460731, 1461601, 1464559, 1464733, 1466473, 1466821, 1468387, 1474129, 1476217, 1479871, 1489093, 1489441, 1501447, 1504057, 1504231, 1514323, 1514497, 1516759, 1519891, 1520587, 1532767, 1543207, 1548949, 1555039, 1561303, 1563217, 1575919, 1583053, 1586881, 1589317, 1597147, 1603237, 1608283, 1612111, 1614721, 1615591, 1646563, 1646737, 1663267, 1686409, 1690933, 1693891, 1694761, 1695283, 1695457, 1709377, 1711117, 1711639, 1718947, 1728691, 1730431, 1732519, 1735477, 1738783, 1739653, 1748179, 1759663, 1780717, 1784023, 1788373, 1809079, 1813081, 1813429, 1814473, 1815343, 1815691, 1817083, 1822477, 1822999, 1830307, 1833613, 1837789, 1841443, 1845271, 1848577, 1852057, 1866847, 1873633, 1877461, 1881463, 1883551, 1891381, 1893643, 1906867, 1907911, 1915567, 1916611, 1923049, 1927399, 1931227, 1935751, 1950367, 1951237, 1951759, 1967071, 1975249, 1981513, 1987429, 1991779, 2003611, 2008309, 2008483, 2011441, 2013703, 2014921, 2022751, 2028319, 2042587, 2046937, 2066599, 2071819, 2074081, 2074951, 2085739, 2089393, 2094439, 2101051, 2103139, 2107837, 2114797, 2116537, 2117233, 2118973, 2130979, 2133589, 2141941, 2146987, 2147161, 2157427, 2163691, 2171347, 2201623, 2207539, 2221111, 2233117, 2235553, 2267047, 2271571, 2279749, 2295583, 2309677, 2312983, 2316463, 2333167, 2337343, 2349001, 2350219, 2351263, 2362573, 2362747, 2365357, 2376667, 2379799, 2401549, 2404159, 2417557, 2426779, 2430607, 2461231, 2461579, 2463319, 2463667, 2465059, 2478631, 2479327, 2480893, 2491681, 2496379, 2503339, 2507689, 2507863, 2521609, 2524393, 2532919, 2536921, 2538313, 2546317, 2558323, 2579377, 2589121, 2594167, 2603563, 2614351, 2619223, 2621137, 2636449, 2643757, 2678383, 2689171, 2689867, 2692303, 2719447, 2729017, 2729539, 2733541, 2758771, 2759467, 2760337, 2765731, 2768863, 2774953, 2800531, 2803663, 2819497, 2825239, 2842291, 2844379, 2857951, 2863867, 2865259, 2867869, 2890141, 2891011, 2909107, 2913457, 2918329, 2928943, 2945647, 2951041, 2980969, 2987407, 2989321, 2997847, 3008113, 3015247, 3057007, 3058921, 3071623, 3078409, 3084847, 3101029, 3104509, 3104683, 3107989, 3115993, 3130957, 3141223, 3142267, 3150619, 3166453, 3167497, 3170281, 3177067, 3183679, 3184549, 3187159, 3196033, 3200557, 3202123, 3202993, 3203341, 3208039, 3220219, 3224047, 3229267, 3259717, 3264589, 3267547, 3269809, 3289123, 3334189, 3342541, 3352633, 3352981, 3361507, 3376297, 3388477, 3417013, 3426583, 3477217, 3548731, 3554821, 3556387, 3574657, 3575701, 3585097, 3588229, 3597277, 3617983, 3643561, 3666529, 3682363, 3694891, 3698719, 3709681, 3730561, 3741523, 3750223, 3761707, 3781543, 3785371, 3785893, 3787981, 3794941, 3817213, 3821737, 3824173, 3853753, 3864019, 3889423, 3894469, 3908911, 3926137, 3936577, 3952759, 3992083, 3994171, 4010353, 4010701, 4030363, 4041151, 4042891, 4055071, 4075429, 4075777, 4082041, 4084999, 4096657, 4100659, 4104313, 4108141, 4134589, 4143637, 4144681, 4157731, 4200013, 4218631, 4219849, 4249951, 4253257, 4261261, 4262653, 4263001, 4278139, 4281619, 4308589, 4311547, 4332079, 4337821, 4364791, 4379233, 4383061, 4427083, 4429171, 4430563, 4450399, 4463971, 4482589, 4490767, 4493551, 4513213, 4514431, 4536529, 4536703, 4548013, 4568719, 4584379, 4591861, 4603171, 4608391, 4626313, 4642843, 4644061, 4664593, 4681471, 4682863, 4695391, 4738891, 4811101, 4821541, 4829371, 4863823, 4876873, 4882789, 4896013, 4898623, 4926637, 4964569, 4972573, 4989973, 5033647, 5043217, 5066881, 5104291, 5124649, 5128999, 5137003, 5151967, 5185027, 5189551, 5236183, 5237923, 5249581, 5276377, 5279509, 5298823, 5304913, 5306479, 5329273, 5332753, 5352937, 5379559, 5384779, 5385997, 5424103, 5424799, 5427061, 5427757, 5437153, 5462209, 5466211, 5481523, 5505187, 5519803, 5537029, 5537377, 5553907, 5557213, 5569567, 5577223, 5586793, 5594101, 5601061, 5626987, 5678143, 5694673, 5711203, 5742523, 5743393, 5797159, 5820823, 5838919, 5842399, 5866759, 5871457, 5878417, 5898601, 5902081, 5906779, 5908519, 5925223, 5932357, 5935837, 5968723, 5986819, 6015007, 6020923, 6039193, 6045631, 6054157, 6059377, 6067903, 6071383, 6088087, 6089131, 6124801, 6130021, 6130717, 6149161, 6157513, 6170389, 6194053, 6209191, 6221023, 6247471, 6269569, 6298801, 6342997, 6346651, 6374491, 6393109, 6400591, 6459577, 6484981, 6502207, 6522739, 6538747, 6569371, 6582073, 6598777, 6611653, 6629923, 6656371, 6733453, 6738499, 6743197, 6749983, 6773647, 6784087, 6799921, 6802183, 6828457, 6865519, 6868999, 6893011, 6906931, 6935293, 6947821, 6988537, 7034299, 7040041, 7200643, 7244143, 7256497, 7270591, 7272679, 7283989, 7287817, 7309741, 7326967, 7335667, 7344193, 7382821, 7401787, 7434499, 7455379, 7475389, 7495051, 7531417, 7539943, 7596493, 7605193, 7622767, 7638601, 7645387, 7676359, 7678273, 7709419, 7720729, 7733431, 7776931, 7807729, 7828783, 7831567, 7944667, 7986079, 8026969, 8029579, 8034973, 8040367, 8109097, 8136067, 8148421, 8169823, 8260999, 8268829, 8285533, 8314069, 8391499, 8403853, 8419861, 8456053, 8471713, 8526871, 8534353, 8572633, 8577853, 8581681, 8619439, 8642059, 8651977, 8703307, 8720707, 8726449, 8746459, 8812231, 8814667, 8817103, 8827543, 8846683, 8878873, 8911933, 8932813, 8978923, 9072361, 9131347, 9163537, 9188593, 9234529, 9292471, 9295081, 9310393, 9380341, 9390607, 9392869, 9409747, 9420709, 9455161, 9498661, 9512929, 9520411, 9539029, 9560779, 9586531, 9610717, 9628639, 9639601, 9648997, 9863713, 9889813, 9917653, 10015963, 10017529, 10028839, 10077037, 10079647, 10095829, 10137937, 10148029, 10187701, 10193269, 10195879, 10200403, 10233637, 10305151, 10348477, 10372837, 10399633, 10417207, 10450963, 10468363, 10549969, 10620439, 10730059, 10737193, 10765207, 10777909, 10863343, 10871869, 10876741, 11001673, 11081017, 11096329, 11147833, 11201251, 11214649, 11263717, 11273461, 11304607, 11319223, 11382211, 11382733, 11409877, 11527501, 11575873, 11581441, 11588749, 11609803, 11627899, 11628769, 11644951, 11666701, 11795809, 11808337, 11945449, 11976073, 12002173, 12035407, 12091957, 12106921, 12218629, 12225937, 12239509, 12287533, 12367573, 12380623, 12550273, 12768121, 12770209, 12867301, 12898621, 12991189, 13000411, 13012243, 13018333, 13100809, 13206253, 13283509, 13292557, 13406527, 13576177, 13587487, 13590271, 13699717, 13715899, 13741477, 13774363, 13831957, 13842397, 13928701, 13965937, 14123059, 14146723, 14190571, 14209537, 14247469, 14277223, 14282791, 14401633, 14410681, 14431909, 14479411, 14563453, 14632183, 14652193, 14655499, 14678641, 14753287, 14821669, 14834893, 14836981, 14895619, 14930767, 14942251, 14963653, 14980183, 15004369, 15009067, 15012199, 15025597, 15075883, 15098851, 15112249, 15115207, 15142177, 15196639, 15266239, 15318091, 15324181, 15389953, 15473473, 15518539, 15626593, 15662089, 15687493, 15712549, 15785281, 15842353, 15919087, 15954409, 16029751, 16033753, 16067683, 16172953, 16213147, 16215061, 16248643, 16310761, 16392889, 16452571, 16558537, 16594729, 16642231, 16812403, 16816231, 16987447, 17059657, 17069401, 17094283, 17229481, 17273329, 17287423, 17464903, 17513449, 17524063, 17560429, 17683969, 17776189, 17866843, 17898337, 17907733, 17945143, 17987773, 18179521, 18219193, 18375967, 18401023, 18429907, 18583897, 18713701, 18767467, 18809053, 18844549, 18918499, 18993841, 19047607, 19048129, 19055089, 19102243, 19243009, 19272937, 19316959, 19389691, 19524541, 19549597, 19732123, 19760833, 19770403, 19778233, 19873933, 19970329, 20016613, 20029663, 20068117, 20117707, 20190613, 20279179, 20330857, 20363743, 20438389, 20465707, 20636401, 20649973, 20697997, 20701651, 20705653, 20758201, 20846941, 20960911, 20966131, 20980747, 21110029, 21189547, 21241573, 21309259, 21309433, 21335359, 21341797, 21449503, 21518059, 21603493, 21624373, 21656911, 21811771, 21870931, 22064767, 22070509, 22248511, 22288009, 22310107, 22311673, 22417987, 22777819, 22878043, 23041429, 23071879, 23112073, 23172973, 23289379, 23472601, 23540113, 23616499, 23776579, 23881327, 23884459, 24150157, 24245161, 24281701, 24285529, 24345907, 24397237, 24480583, 24536959, 24657019, 24729403, 24768553, 24776383, 24789781, 24878521, 25155703, 25189981, 25258537, 25279417, 25293511, 25294903, 25296121, 25558861, 25628983, 25775143, 25914517, 26139151, 26180389, 26327941, 26383969, 26430949, 26518123, 26730229, 26760157, 26865079, 26959039, 26959213, 27004801, 27144001, 27192199, 27323221, 27488347, 27508357, 27539851, 27904729, 28145371, 28159639, 28224019, 28471969, 28562623, 28717309, 28721311, 28903141, 28990141, 29102371, 29104633, 29141521, 29197897, 29251489, 29466553, 29811073, 29819599, 29938789, 30231283, 30297403, 30471751, 30647839, 30771031, 30899269, 30928501, 30986443, 31093627, 31103719, 31114333, 31217167, 31574041, 31594573, 31969543, 32216623, 32350777, 32451697, 32786821, 33102109, 33239221, 33311779, 33327961, 33370591, 33437581, 33521623, 33653341, 33799501, 34024657, 34031443, 34380487, 34451653, 34466269, 34714219, 34796869, 34872037, 34887871, 34972957, 34980091, 35028811, 35048647, 35100673, 35185237, 35632243, 35668783, 35696797, 35837041, 36173209, 36181039, 36231847, 36471619, 36871819, 36893917, 37151089, 37334833, 37354843, 37716589, 39109459, 39274933, 39514183, 39669913, 39672001, 39705583, 39805807, 40690249, 40792387, 40815007, 40927063, 41408347, 41743123, 41912077, 41996467, 42047623, 42116353, 42137059, 42154807, 42155329, 42283741, 42557791, 43034899, 43443973, 43488343, 43493041, 43514791, 43594483, 43609099, 43720459, 43744993, 43805197, 43898461, 43959709, 44094907, 44339551, 44689639, 44692771, 44807089, 45422527, 45631849, 46028569, 46117831, 46342291, 46527427, 46673239, 46807219, 46841671, 47115547, 47214901, 47238391, 47274757, 47511397, 47552809, 48254377, 48616993, 48683809, 48768373, 49120723, 49216423, 49360147, 49408693, 49671781, 50032657, 50148193, 50168029, 50361517, 50386573, 50590849, 50847673, 50868379, 51236041, 51713149, 51926299, 52215661, 52391749, 52722697, 52897567, 52974127, 53260357, 53400079, 53519269, 53728243, 53820463, 53850217, 53900851, 54282433, 54503587, 54518899, 55280497, 55429963, 55459891, 55630063, 55737943, 55823377, 56625343, 56964469, 56982043, 56999443, 57078787, 57130639, 57156739, 57187189, 57926689, 58068847, 58129399, 58200217, 58614337, 59387071, 59504521, 59785879, 59873053, 59981629, 60046531, 60686851, 60880513, 61495081, 61615663, 61656379, 62245021, 62380741, 62713081, 63295807, 63435877, 63620143, 63763519, 63777613, 63826507, 63937693, 64270033, 64593847, 65936083, 66098947, 66312619, 66441379, 66528379, 66600241, 67361839, 67601263, 67718713, 67887319, 68662141, 68821003, 69054859, 69076261, 69359707, 69664381, 70189513, 70391179, 70448251, 70459387, 70928839, 71034283, 71446663, 71599261, 71744899, 71764909, 71806321, 72139009, 72214699, 72447511, 72482659, 72692329, 72836053, 73203019, 73241647, 73620619, 74889427, 75407947, 75510607, 75729847, 76625077, 76747921, 77296543, 77326819, 77352049, 77864827, 77917027, 78657049, 79187749, 79840597, 80206171, 80805253, 81173959, 81382933, 81531007, 82208911, 82226659, 82429717, 82679059, 82931533, 83545057, 83590471, 83653111, 83894971, 83940559, 84301783, 84305959, 84341977, 84870067, 85329253, 85406683, 86011333, 86190553, 86583097, 87023143, 87305719, 87349219, 88004503, 88451683, 88776019, 89058421, 89219371, 89517433, 89634709, 89653327, 89744329, 90013681, 90977989, 91069861, 91455097, 91464319, 92650303, 93267481, 93348043, 93589207, 93830371, 94018291, 94049611, 95617351, 95694433, 95849641, 96236443, 96676837, 97116883, 97165603, 97746763, 99378709, 99487459, 99996931, 100318483, 100353283, 101284879, 101449483, 101458183, 101777821, 102423187, 104334229, 104509621, 105144199, 106049869, 106321309, 106768663, 106845049, 106866103, 107705827, 108322309, 108881023, 109569193, 109611301, 109774339, 110262931, 110481649, 110869669, 111136933, 111179389, 111331291, 111385231, 111764203, 112332487, 112399129, 112545289, 112844047, 113377357, 114432841, 114563167, 115138237, 116078881, 116477167, 117704911, 117706651, 117761983, 118597009, 121256251, 121405717, 121543003, 121666717, 122818771, 123102391, 123197047, 123700429, 124368763, 125024743, 125841151, 126724201, 126806677, 127131709, 129346207, 129589459, 129860551, 132944353, 134190019, 135030787, 135194347, 135259249, 135511897, 136096711, 139379917, 139590109, 139786033, 140515093, 140632543, 140882059, 141134011, 141458869, 141485317, 142338613, 142689049, 143328151, 143349901, 143442121, 143970907, 144907723, 144930169, 146622319, 146657989, 146941957, 147155803, 148749817, 150217681, 150243781, 150684349, 150785617, 150817633, 151206697, 151291087, 153015427, 155503279, 155722867, 157645219, 158280841, 159778807, 160244779, 160668643, 160742941, 161066233, 163778023, 164278969, 164293237, 164360227, 164665597, 164669773, 164868133, 165189859, 165485311, 166321381, 167306221, 167591929, 168585817, 169746571, 169924399, 170471281, 171907303, 172091743, 172637581, 172726843, 174548971, 175457773, 175636471, 175681711, 177505231, 179431237, 180264871, 181486003, 181828783, 182266219, 182682601, 182967439, 184374403, 186675901, 186746023, 187495963, 189553339, 191070793, 193090759, 194083429, 196827409, 196924153, 196940683, 197384731, 198608647, 199095151, 200228761, 200938333, 200968783, 201798763, 202014697, 202748803, 204829843, 205036729, 205413787, 206351473, 206832757, 208133059, 208597813, 209144347, 209334703, 209495827, 210872863, 211577911, 213200461, 213501133, 214269169, 215864053, 216827491, 217070221, 219828643, 220085119, 221142343, 223487863, 224058061, 224248417, 224458261, 225111457, 225325651, 226089859, 226116307, 226864681, 226904179, 227206069, 227259139, 230393749, 230910703, 231358927, 234103603, 234527119, 234852847, 235498561, 235620883, 236878903, 237048727, 237233167, 237875749, 238703293, 240048139, 240216049, 241498429, 242022343, 242527639, 244680367, 244907959, 245352703, 245425783, 245644153, 246878161, 247796011, 251064949, 252672361, 252843403, 252864979, 253674427, 254199559, 254773933, 255150817, 256421887, 257290843, 257499643, 257915851, 260273203, 260737957, 260746831, 261206017, 262151881, 262274377, 262837789, 263114623, 263282011, 263307937, 265059247, 265315723, 266736607, 267512821, 268258933, 268303999, 268802857, 269596993, 270000499, 272857927, 273195487, 273381319, 275636359, 279239203, 279312283, 279923719, 280271371, 281559319, 282547117, 285126493, 285776731, 286668133, 294321871, 294573649, 294950881, 295680811, 297757501, 298375897, 298637593, 299049973, 300210901, 301485799, 303039967, 303715957, 304597963, 306055213, 307566403, 308909509, 310053559, 310355623, 311698903, 313336417, 314810719, 315444949, 316839907, 318377197, 318514657, 320745163, 322341787, 322720759, 328704097, 329361121, 329696593, 330584689, 333543211, 334481593, 334667251, 336406207, 336507823, 337583143, 338477677, 339225181, 339456427, 340637191, 341770801, 342871003, 343251541, 346031887, 352734367, 353254801, 354409117, 358965307, 359804161, 365251927, 365579047, 367318177, 369185023, 369837697, 373694929, 374802091, 375361849, 375914821, 377722333, 378091213, 378689947, 384224713, 384277087, 386407369, 387521317, 389035291, 391911859, 392238631, 393064783, 394139233, 396512419, 397844563, 398690203, 401576167, 404313883, 405081223, 405931909, 407059777, 408207829, 411907417, 412524421, 419024017, 419861479, 427099531, 427982059, 429442441, 435289189, 443886007, 447369487, 448658827, 449034841, 449628007, 454452679, 459049411, 459236287, 459827539, 460891897, 470994859, 471580717, 477361519, 478419091, 480674653, 481023349, 485948941, 493306531, 494594479, 495422023, 496357969, 498780397, 500950873, 503130919, 507607069, 509382217, 516466453, 516816019, 519427933, 520001263, 522198709, 522849643, 527939317, 528128977, 530721403, 531835699, 536451919, 537816949, 543107767, 543240007, 544105657, 544243291, 545122687, 546151897, 555291943, 556626349, 558478579, 563152393, 566738533, 567204331, 571090969, 571405213, 572693509, 574089511, 576821659, 578606029, 579292981, 579469069, 579593479, 583486207, 587383633, 590634823, 599087221, 601113277, 609542359, 611368663, 612648433, 613571677, 620016811, 622209037, 638055043, 638675701, 640415353, 640473991, 643000471, 643137409, 644316259, 645155113, 648524971, 652417873, 655415023, 663682807, 670989937, 671893171, 675445033, 676977799, 682249651, 686811757, 688661203, 692511997, 694762339, 695016901, 698878309, 700968223, 705937663, 710160469, 710953387, 712247947, 712452919, 713723293, 713758789, 716261257, 717210601, 725255143, 729753217, 732066373, 732385663, 737104369, 738949117, 739121377, 739178449, 745833427, 749437141, 750477487, 754279909, 755507653, 757185361, 757404253, 757889017, 768478831, 775083349, 780576007, 787771951, 788053657, 788062879, 789251821, 791511037, 793140373, 796581571, 801874999, 803344081, 804095587, 813839239, 817234153, 822089623, 822233173, 828037813, 833063629, 837721957, 845071021, 847121089, 849120523, 849797383, 850834597, 854405077, 858790573, 862617529, 867557737, 869785459, 870675469, 879086107, 881382559, 896782429, 897016111, 902967781, 904001863, 904060501, 911035291, 912284437, 917207419, 920455651, 934495363, 935614531, 935735113, 937697311, 938109343, 946744963, 947104099, 949118497, 952204039, 954655699, 956801641, 957342259, 960348457, 965113969, 967485763, 968791111, 974389909, 975015787, 975369181, 985798741, 995577367, 999131317, 1000710889, 1003353427, 1006072351, 1008571513, 1023352987, 1025938453, 1028100751, 1032005833, 1046995933, 1047865759, 1054600081, 1054707961, 1058173867, 1059712201, 1062240247, 1066339513, 1085147347, 1085430967, 1087382551, 1090148803, 1105213897, 1107420043, 1107679129, 1114444423, 1117802971, 1126379431, 1127611699, 1130349589, 1130352547, 1132011289, 1135799617, 1144925569, 1149146983, 1149783997, 1164762439, 1166374201, 1167436297, 1175104303, 1176278281, 1181498803, 1182561421, 1187495887, 1192040419, 1194024541, 1196662729, 1198186621, 1198325647, 1198384807, 1200876661, 1202988499, 1208524309, 1210848253, 1215989779, 1218812233, 1219250017, 1221299041, 1222322161, 1226111881, 1228165777, 1229318179, 1258787689, 1263855787, 1265100409, 1269140167, 1275646549, 1281348007, 1287014143, 1289353399, 1289975101, 1290623251, 1291026931, 1306633687, 1308422407, 1310358157, 1317070207, 1325979703, 1332126427, 1332278329, 1332854443, 1333378009, 1336887241, 1338687271, 1351705603, 1357414543, 1359730657, 1364681653, 1365791599, 1368094489, 1369243237, 1383323143, 1383450337, 1385738611, 1393349719, 1397091589, 1399744393, 1436911837, 1453395379, 1455344179, 1458203521, 1460692939, 1474336453, 1484084977, 1490338537, 1507627351, 1508553727, 1515017653, 1525047709, 1527421939, 1532601223, 1537750927, 1551118651, 1554623011, 1555467607, 1556344567, 1559926531, 1567037041, 1576616959, 1593867493, 1594977091, 1627631497, 1637787181, 1642677277, 1654339279, 1658340583, 1659141157, 1668501487, 1700417959, 1705376611, 1710415303, 1724831377, 1735462081, 1742469409, 1758856903, 1769963323, 1774699777, 1796851369, 1799606659, 1815027931, 1836360157, 1837200229, 1843823539, 1865045449, 1878001141, 1880650987, 1882693921, 1884632803, 1887100123, 1887884863, 1900720321, 1911906433, 1913447203, 1934338687, 1934715397, 1964347423, 1968872293, 1969444579, 1978546693, 1987937473, 1999623139, 2011121929, 2018843353, 2019636793, 2020488523, 2022735907, 2038199113, 2038545547, 2041864771, 2059106083, 2059216921, 2065714429, 2079641737, 2079730999, 2116585243, 2117658997, 2119244833, 2119290769, 2121196417, 2126493673, 2139542281, 2141879449, 2159368537, 2160037219, 2172680929, 2179094917, 2188190419, 2196561559, 2208366241, 2238489817, 2242887841, 2252751901, 2253503233, 2265333493, 2265761881, 2288091301, 2311021717, 2377186783, 2381111353, 2389178167, 2394113851, 2406770437, 2408931517, 2410277059, 2416008097, 2421506671, 2445527719, 2455882111, 2470500547, 2481179101, 2486591197, 2495656249, 2499226903, 2504012947, 2523026449, 2524270549, 2528546773, 2562209509, 2565134623, 2576217031, 2595624469, 2606648413, 2658365911, 2664202393, 2675660989, 2711159947, 2715148201, 2741786209, 2748727243, 2769902347, 2772803623, 2777850841, 2782510039, 2792070991, 2798553361, 2812149199, 2832379657, 2834747449, 2841856219, 2899285267, 2904043993, 2907476839, 2933174551, 2938043593, 2940923641, 2947852321, 2949901519, 2976821233, 2986990663, 3003222427, 3033413689, 3051181699, 3055091827, 3074036599, 3084043513, 3093998923, 3121700419, 3124415341, 3137699371, 3138511081, 3140714443, 3160196701, 3161087581, 3181047817, 3203112061, 3205862479, 3208576009, 3244645861, 3249815749, 3256897201, 3258502873, 3262286329, 3270183667, 3319394521, 3322003651, 3322524781, 3333139129, 3348911011, 3358681981, 3368279647, 3418723813, 3422005801, 3434578519, 3451649659, 3466426087, 3467419453, 3478160299, 3481959937, 3486632011, 3499781713, 3520452391, 3523436317, 3537911203, 3564092461, 3638930731, 3662769427, 3682880869, 3703296463, 3713383417, 3729238819, 3736056661, 3753008437, 3754816123, 3785191999, 3793137883, 3804197149, 3810391027, 3836612827, 3900000331, 3913338823, 3914251801, 3920159797, 3937684033, 3967846933, 4016653237, 4026106657, 4043060347, 4063092967, 4065475027, 4099721533, 4122981157, 4173144487, 4241240431, 4262495749, 4280009893, 4310430139, 4340795749, 4355953759, 4359168409, 4449501031, 4493834491, 4521784459, 4528276399, 4567715587, 4586384743, 4602168283, 4603822849, 4612970377, 4613221981, 4614320443, 4662207331, 4697449987, 4712294971, 4716205621, 4717336969, 4733329483, 4768846189, 4779716839, 4820791627, 4831725961, 4834329349, 4890298537, 4908269953, 4910215447, 4929155173, 4944724867, 4974925003, 5003155459, 5003353471, 5025937801, 5027472133, 5035886599, 5065290337, 5069427361, 5091936697, 5115158563, 5130046699, 5137396807, 5137698871, 5140942231, 5195746837, 5296839619, 5338406653, 5346799891, 5351596897, 5357222317, 5368580689, 5389544383, 5395900603, 5399292733, 5446558267, 5503334467, 5533719043, 5552291629, 5554966357, 5589300559, 5591358979, 5605345099, 5658132349, 5768868037, 5811512131, 5823488899, 5841928549, 5867071549, 5893414627, 5894059471, 5993190751, 6014303041, 6025955647, 6026752741, 6047417503, 6077564743, 6095570263, 6169741243, 6200751871, 6248152429, 6298704301, 6331618663, 6341972881, 6362336623, 6366566737, 6390084577, 6430023493, 6446705569, 6447540943, 6477645379, 6683842687, 6704932183, 6724582873, 6779973163, 6821356627, 6864330799, 6882374251, 6926931127, 6940101361, 6980809357, 7017899719, 7036629079, 7098762043, 7099829707, 7131918439, 7155317437, 7181961187, 7246378771, 7246791499, 7256472859, 7294926511, 7305184507, 7307330101, 7405029361, 7454414389, 7572515149, 7665913477, 7741240687, 7779859291, 7783985527, 7803682501, 7867009279, 7955278087, 7997020513, 8069471677, 8076580273, 8099105617, 8156346571, 8233041247, 8328392377, 8331543169, 8335271641, 8344218721, 8357178763, 8380079947, 8386739971, 8417771827, 8437178917, 8483725309, 8492254093, 8526322771, 8528133067, 8579861701, 8597878183, 8603302633, 8625249949, 8630128561, 8634895987, 8658402691, 8787682081, 8825121139, 8835538519, 8877272419, 8880607129, 8916630523, 8928553351, 8953354789, 8992906903, 9016373239, 9028915681, 9070066507, 9104857633, 9117798187, 9157148461, 9160086277, 9175675981, 9222693913, 9312800857, 9373271251, 9415689841, 9418978441, 9468804559, 9470833921, 9654161191, 9784060021, 9817279231, 9862658257, 9892136119, 9972553873, 10015485241, 10126166293, 10158135313, 10179018097, 10207124143, 10232563987, 10306678939, 10339861087, 10350656047, 10369694779, 10386810289, 10480902877, 10565964691, 10589595283, 10628618437, 10633743259, 10775291911, 10864318837, 10875191401, 10975757137, 11015991331, 11126624533, 11126981233, 11280826987, 11287246717, 11347387903, 11381122327, 11422108549, 11479421017, 11492382973, 11495059789, 11499361591, 11504216887, 11590761529, 11648916157, 11668785217, 11719831423, 11797486927, 11931732277, 11939013133, 11957538739, 12025092151, 12053727331, 12058587847, 12145120657, 12154859263, 12346520437, 12373546639, 12411519007, 12530854471, 12550130539, 12614750311, 12705722209, 12786427933, 12956063143, 13048202929, 13125352441, 13138325533, 13169135191, 13194937477, 13196203849, 13239208819, 13309806187, 13413653389, 13530463417, 13622939719, 13634063713, 13681640881, 13710036463, 13747802119, 13765531501, 13819164217, 13928248123, 13980171637, 14084881357, 14169358183, 14200815121, 14209652581, 14285366419, 14401975999, 14434411513, 14518078891, 14529890707, 14552092411, 14565746539, 14853935431, 14967037171, 14970420427, 15076897291, 15175358323, 15231688387, 15327316177, 15354599899, 15358183777, 15444259141, 15565719493, 15656025841, 15688632397, 15747602389, 15919815757, 16022076601, 16039342447, 16078548649, 16225278151, 16225896199, 16235114023, 16346378149, 16423126417, 16423911331, 16494424831, 16604673493, 16727937703, 16752417589, 16802840701, 16885878199, 16947435919, 17068090651, 17072613781, 17079696973, 17166557251, 17250995623, 17296126699, 17348958667, 17388131461, 17424044017, 17711402407, 17717284999, 17820405403, 17833170913, 17894210461, 17995202671, 18057782467, 18076186621, 18081550867, 18125077837, 18207901489, 18435361597, 18448155991, 18484432903, 18568181539, 18734858749, 18735565711, 18852229057, 18869064427, 19100476771, 19125350593, 19187273713, 19237857949, 19274584999, 19291893823, 19329818863, 19370280997, 19447215097, 19483788679, 19489719121, 19587752983, 19694508247, 19727717539, 19790792887, 19902733351, 19927653979, 19935840853, 20402688247, 20512673473, 20543306869, 20661445039, 20775343177, 20813230981, 20994382729, 20994492523, 21187667323, 21493521739, 21531039967, 21597413311, 21664140397, 21868792063, 21871141063, 22082417083, 22355184877, 22446738109, 22525232467, 23033244607, 23040088723, 23083400107, 23301752533, 23543841691, 23750750137, 23752845619, 23916215263, 23962927999, 23975179861, 23980880449, 23988287629, 23995350637, 24074579797, 24120994297, 24189340453, 24211930177, 24264570919, 24275896753, 24403045687, 24419007751, 24465034753, 24684903241, 24902649451, 25075790239, 25597302517, 25607772271, 25658552779, 25717218271, 25748716099, 25767924307, 25819757863, 26000023081, 26018513887, 26163022279, 26290319113, 26870496013, 26881302979, 27075435997, 27225915721, 27282769873, 27292126201, 27334184611, 27370889563, 27531984157, 27716855851, 27783685249, 27897858829, 27900337459, 28058599333, 28063024849, 28104744133, 28411186147, 28649221453, 28938972169, 29006984593, 29057024383, 29106001381, 29111352577, 29133580033, 29403646477, 29418754549, 29505054547, 29681148463, 29816410147, 29873108569, 30373671421, 30630231637, 30683341657, 30717823933, 30960031411, 31330672987, 31443903661, 31626827077, 31708656493, 31925581423, 32052257773, 32089349701, 32274871633, 32348393071, 32353536511, 32427814849, 32583809329, 32846293201, 33094156897, 33208852477, 33308058403, 33390223291, 33564508651, 33739200823, 33748414471, 33937441633, 33960890917, 33991111411, 34068800149, 34194777541, 34257998179, 34453533637, 34492528951, 34804544707, 34945738573, 34966528963, 35228428717, 35389348441, 35543692879, 35575952131, 35803792081, 35830456537, 36116382559, 36213883807, 36287084737, 36657374137, 36779903023, 37074876697, 37084787563, 37102872949, 37307037067, 37482069409, 37515342037, 37519743367, 37680074101, 37708559293, 37944923677, 38755304491, 38755647967, 38944446841, 38993017201, 39274913383, 39307570573, 39388065061, 39391261963, 39411119017, 39606707197, 40035993559, 40043923957, 40847383747, 41000121121, 41117479423, 41255316133, 41278726789, 41476971427, 41774386321, 41923099249, 42104834767, 42120250297, 42270513391, 42424532797, 42633404311, 42879397507, 42925606513, 43220773501, 43228455601, 43381690789, 43515143743, 43808308513, 43932074539, 44094431851, 44491852897, 44562136891, 44679110479, 45243432151, 45674826523, 45884185063, 45932742199, 46073771983, 46083801691, 46090709491, 46181711839, 46785968599, 46837466161, 47104659691, 47404469173, 47559278539, 47612575783, 47754749269, 47990645419, 48407338183, 48603397729, 48729844573, 49053421063, 49395045877, 49653057253, 50311713721, 50537552323, 51542473663, 51850891447, 52030210801, 52072940503, 52292471431, 52409290159, 52864708537, 52908773689, 52954683241, 53266265737, 53274672547, 53328287167, 53702457463, 54022502623, 54257229667, 54718418059, 54991441633, 55085804791, 55136878837, 55287443647, 55460311429, 55930284559, 56241627283, 56288808601, 56312460943, 56496838129, 56549811559, 56823836677, 56851843021, 57071964853, 57622675723, 58234257883, 58593215533, 59137687111, 59513726689, 59772970681, 60350882797, 60504330091, 60692446189, 60824259019, 60880124677, 61278558403, 61346109379, 61525774819, 61668375649, 61805963017, 62028644911, 62349344137, 62925706783, 63097365787, 63112858573, 63368823361, 63375863227, 63464461243, 63506592211, 63704040973, 63803243419, 63808641073, 63838880359, 64038285403, 64096931581, 64154828167, 64359071629, 64391781541, 64487977789, 64824061573, 64992857581, 65333681821, 65512526503, 65583431503, 65674999003, 66212944537, 66301199773, 67013457289, 67129859287, 67326603523, 67633967563, 67955476933, 68376181963, 68412258427, 68661746761, 68851213099, 69061472959, 69236519221, 69249693979, 69279846787, 69439019203, 69697316809, 69765627469, 69765825133, 70303429279, 70366764931, 70459111603, 71042149411, 71226162937, 71343992953, 71350953301, 71536785127, 71537616499, 73025995621, 73288635919, 73487951701, 73558412131, 73620030577, 73865890141, 74347915207, 74794937389, 75429119713, 75508562893, 75737096059, 75826470289, 75842106973, 76183047271, 76349915359, 76831543183, 77353408507, 77506381651, 77684836051, 78315413443, 78427819879, 79054652617, 79234303963, 80276008903, 80347048927, 80961172531, 81513786961, 81835662949, 82008802867, 82326431167, 82586478343, 82610787709, 82680625393, 83047252441, 83466199027, 83953239817, 84207334801, 84396290623, 85211772421, 85229181991, 85391185909, 86725294543, 87230282389, 87729122989, 87767628493, 87773396593, 88294131787, 88436138407, 89111166883, 89761251241, 89802074077, 90310788133, 90431830711, 91112646253, 91302030637, 91539989731, 91744860463, 92588062027, 92774133799, 93115796023, 94092414001, 94258115071, 94309484743, 94607922931, 95138698273, 95206533913, 95416434811, 96519851809, 96533882647, 97079070931, 97255861369, 97561729357, 97609186639, 97835599963, 98046372427, 98116046551, 98354870599, 98795072503, 98892642481, 98928350413, 99664049209, 100597663609, 100610003167, 101404924603, 101408873707, 101504954071, 101510820133, 101813685577, 102309937753, 102319891423, 102985890691, 102986656639, 103839487537, 104717389573, 106437730879, 106835628511, 107149238629, 108508994863, 109322988787, 109442407423, 109453503403, 109996125709, 110182571929, 111198788131, 112522069789, 113398149349, 113518515241, 113885089741, 114288758953, 114702368089, 115065942307, 115140801631, 115330940479, 116266888741, 116288678761, 120661090309, 121541568937, 122804178187, 123072848629, 123319715131, 124359303883, 125356979863, 125816261737, 127010747377, 128031877513, 128260564843, 128562590911, 128602033927, 130316724757, 130367028331, 131813589511, 132195837409, 132582228073, 133088059297, 133531574161, 134666274619, 135150025939, 135832288987, 135862727503, 135905285119, 136365146413, 136674520501, 137017641193, 137172632911, 137898203341, 138217728937, 139157782207, 139537488661, 139706469109, 141328461787, 141925477363, 141929763853, 142018650013, 142440531979, 143220774079, 143310434887, 143497914493, 143674629937, 143681212009, 143709626383, 143989141027, 144007543093, 144040138339, 144597643039, 145124469103, 145555957957, 145647490831, 146093287531, 147325270693, 149438010013, 149815680319, 150139030087, 150181367767, 150392076199, 150559381201, 150850166347, 151249312429, 151694356927, 152009428993, 152574216811, 154707209731, 155574211189, 155710618837, 156200963713, 157010561353, 159360870007, 159811277707, 160790538049, 161721621967, 161840239681, 162259916023, 162279354607, 162774089329, 162803242507, 163673221627, 164265876241, 164481940393, 165050855143, 166338119671, 167471962831, 170913261229, 172297888849, 173039491987, 174464610973, 176788152511, 180220492519, 180408638197, 181317167191, 181360851979, 181836400939, 182404746187, 182473521427, 182798782237, 183522917167, 184869292627, 185146826629, 186403084009, 186622731343, 186794519629, 189268281283, 191947473079, 192454200751, 193585217629, 193941034579, 195342858619, 195434196439, 195643479289, 195670864453, 195737006029, 195739303699, 197254642729, 198106694629, 198404684767, 200120500507, 200495317039, 201036963031, 201801734269, 202063599571, 202532859649, 203212232731, 204062118679, 205104149347, 205228351243, 206064170161, 206119141981, 207114867073, 207235547557, 209045086483, 209251379317, 209779730491, 210773943871, 211619765527, 212199905017, 213256454767, 213653792467, 214290335971, 216630580813, 217278791539, 218552499379, 219369276433, 219592847293, 220726116427, 223157733541, 224154227887, 226895084761, 229480701271, 232887662509, 232966741333, 234158639419, 235385884387, 236895551887, 238681682593, 240929522299, 241036072591, 241506969313, 245903306731, 246282256111, 249967019311, 250777254313, 251660144059, 251898644119, 252636522613, 253935251653, 257262733867, 257971274917, 259519881877, 261920262859, 262804854673, 266312808469, 268477838617, 269172673513, 272263149283, 273778285603, 274595251621, 274894821679, 276050258413, 279978358351, 281804880199, 286176670393, 287130731011, 288226172023, 290258312629, 293711759881, 296322241339, 297467562583, 297740516383, 299660681899, 303455634949, 304309791553, 305157957859, 305919176539, 307514990221, 309082914313, 309822285901, 319491426403, 320370629611, 321688560073, 330900200461, 332654904157, 332782605541, 341560447747, 342704075101, 343410567301, 343917880483, 344296156657, 345358391509, 345929394781, 346557272563, 349586689471, 353223711073, 354337510129, 354796579201, 355183284979, 358857557197, 359915520001, 360136202113, 360372019789, 360509989087, 362318131663, 362323023499, 362829816073, 363220559521, 366111953191, 367607895223, 368341427371, 368419158391, 368564489107, 370479157963, 372585680437, 373342005367, 373522068223, 374748087709, 375178848721, 376183338367, 378284806489, 378964020187, 379181655037, 380946795601, 383122604353, 384241487863, 386676528079, 389403074323, 398919172429, 400227993643, 400883612593, 402632916199, 409659529141, 411729974107, 415270345669, 417344546251, 419448005107, 422004030829, 427911040771, 428208260437, 429466825231, 429902307907, 430819954849, 435426526897, 435504202411, 436472080021, 439462608799, 440396804011, 444696288331, 445019089129, 446972962273, 451559430013, 452244010567, 456626606509, 457571361433, 457834556617, 458986981759, 468613737253, 474848326207, 484370428357, 485197828459, 488991575341, 490287905269, 492110357953, 492382253137, 493080067087, 495324259753, 499789882927, 500107301557, 500440013743, 501593245201, 504664598371, 510944740699, 520133973889, 527278379611, 529543014493, 533285953723, 534178251823, 559293235561, 560434769173, 560684193127, 563283753649, 566751362413, 570947857177, 575549152327, 585331489399, 587487531577, 590122684147, 591005737453, 591715186609, 593016452569, 596064655561, 596212270897, 597834932953, 603460641967, 605730515641, 609764731393, 613673063539, 614921678143, 617118389341, 623025806791, 631378104157, 633361483873, 633650135779, 636480186967, 643471201249, 655615152277, 656360842327, 660463192303, 665088195649, 667615220617, 670514453683, 670917074197, 672109924933, 673504528321, 683677557337, 686809316521, 691084092319, 691397249341, 692611370359, 693135648367, 700530766861, 701611221601, 703081811833, 706982202967, 707615289961, 708978201859, 714279097591, 723066298039, 727547116459, 733441952491, 736733212951, 745510620853, 769355914759, 773014524367, 773849453623, 776974255591, 778686459787, 783052138753, 785163019057, 786107760409, 789198781843, 792107594479, 796583823823, 799135671979, 819440329843, 823899035647, 832904882581, 833653348453, 836032368151, 846724733227, 848308253113, 857474315767, 871358084791, 874569644551, 881339538241, 885011126683, 885986543539, 887401699981, 893112065389, 893865204553, 897233100007, 899158005457, 902717854231, 904429924969, 904455945451, 905234846881, 910562865559, 912468928201, 917957282833, 920858268949, 922762088557, 926506563511, 940635859759, 945803831671, 951926224729, 960089967691, 966210946651, 971919718741, 972329862319, 972802907767, 972927134197, 981616104163, 989757764089, 998560416781, 1000210222489, 1008159893599, 1019741048587, 1025101771261, 1025840070139, 1027949144149, 1030424175307, 1036735159309, 1041223284337, 1042788248863, 1043310089827, 1045991763559, 1057731588973, 1061712760477, 1074734149309, 1091699796937, 1095751618531, 1100672491999, 1104859625041, 1105308077677, 1110611037397, 1119642430141, 1121621159707, 1146349800979, 1156869247813, 1166307468217, 1174399117237, 1187265358099, 1187305560799, 1193084212681, 1194988162441, 1199661450439, 1232795533027, 1243641237169, 1249172048497, 1271794751761, 1285139405971, 1291184779321, 1295322151147, 1296840979573, 1298003255551, 1312478111797, 1336887032071, 1360879139869, 1370026320391, 1370318594629, 1371361589779, 1373199949543, 1377610751929, 1398854547301, 1425617541589, 1426808550709, 1439528194483, 1445424256867, 1453116316801, 1482112896889, 1484469061513, 1489026274633, 1490711725807, 1502919375277, 1506129162673, 1513918883761, 1525155843433, 1527824756179, 1541192925271, 1543033455511, 1552264753027, 1554721708021, 1561531742119, 1567752375577, 1574760206959, 1591565650699, 1594419521791, 1607036272057, 1608276574507, 1609991106127, 1622923052173, 1625573909809, 1636844396149, 1659039440299, 1666830440983, 1672443986701, 1682123655073, 1683665609839, 1695849088447, 1704488126329, 1729540674343, 1751896769239, 1821958574113, 1832266738837, 1834837465297, 1846553954701, 1866764716249, 1868882221429, 1885256801671, 1889577761593, 1934619504427, 1938631540921, 1973873586007, 2009137192129, 2055322869949, 2062431436717, 2069910479587, 2074933436593, 2078629994383, 2085097645201, 2086540769359, 2100702733933, 2108964599323, 2124204164689, 2138486280439, 2143463204353, 2218581829591, 2241202952239, 2245270657423, 2268427571659, 2275903906567, 2343859882183, 2350326257233, 2350480804381, 2361407128291, 2368623926191, 2371700591059, 2381367968593, 2388693624721, 2408755714753, 2416524062899, 2454956680909, 2457015364693, 2482814033929, 2488546432783, 2505691215151, 2526181184929, 2526282595087, 2550038899999, 2554258879369, 2580323164477, 2594941596649, 2620263945583, 2621247578197, 2639786249857, 2665693655173, 2679356490133, 2683492537819, 2712079644751, 2770447771969, 2809813891627, 2818892281423, 2833037149279, 2862966455149, 2889966994237, 2943633389677, 2950926697639, 2963472155233, 2984145959317, 3005790609799, 3007534847047, 3055400631457, 3081739026073, 3120020085733, 3131154437953, 3151541659339, 3199307093077, 3200265070957, 3205539474667, 3245703977491, 3253601301397, 3304926330217, 3325195694269, 3356478521083, 3398293818109, 3403274145811, 3423884319139, 3480591205543, 3502939154107, 3514984683601, 3610295068021, 3612439707109, 3628219928929, 3640131438403, 3663848492221, 3672503234299, 3707869508599, 3728370224443, 3788537274499, 3840949529617, 3845361830491, 3859863577741, 3870355793749, 3894894365773, 3960703000603, 4057747220551, 4071726962929, 4104709804369, 4114442177689, 4118440655233, 4131506423461, 4189737812431, 4211282219251, 4241368867231, 4256169442081, 4309007773957, 4311503203309, 4364227979131, 4384326311797, 4387801528711, 4471247889649, 4509098807737, 4527918119821, 4560664278283, 4561475207893, 4605704242141, 4620659835331, 4639561379989, 4653038761747, 4674998972887, 4676149643239, 4707534017671, 4778613645811, 4820046507889, 4862169685039, 4891478857171, 4958205976753, 4983589131379, 5011782751753, 5103857304979, 5110110931969, 5112161801239, 5203202783329, 5260185331147, 5275366038751, 5298082167661, 5324335061809, 5363593283371, 5463635734033, 5500909365883, 5538919441423, 5641772746549, 5646710503237, 5662801049707, 5737277325583, 5743212569287, 5758020166429, 5829342812191, 5841723270283, 5845675719607, 5848523414917, 5935861049389, 5969804904487, 6088312623517, 6167116806787, 6215238724939, 6283569522481, 6297692479387, 6495874789021, 6521539834087, 6957675904447, 7152272177959, 7158773042767, 7338960841903, 7402879441579, 7464726012019, 7504072181707, 7804827674041, 7834603437439, 8193596951623, 8227124601331, 8249598394699, 8329817733019, 8400882741331, 8435042646661, 8442625397359, 8448047004373, 8451505318231, 8456608655581, 8465751706213, 8563281582433, 8713442938111, 8769293347621, 8795554911667, 9077679232201, 9374731239283, 9494138489419, 9627457204681, 9857737155463, 9991476261337, 10195685203999, 10436960418013, 10553599969513, 10575594901483, 10592938637539, 10616807610061, 10652661455329, 10743017752813, 10914991186123, 10969621064803, 11002739169841, 11256851114491, 11297372941801, 11830910050951, 11922244440889, 12409731520357, 12447460243639, 12527171669869, 12769530242683, 12798789360757, 13252911302689, 13379931110071, 13641849253609, 13721786814937, 13813899425269, 13865162668429, 13901012083657, 14093150732797, 14272087554931, 14406106651861, 14639429617651, 14646639724903, 14692424755369, 15119522083921, 15372196299457, 15379551651991, 15411118036687, 15884881328473, 16148873107789, 16475528673427, 16954689621997, 17252473878847, 17403558641041, 17476764273391, 18004930968763, 18134455781587, 18255098783899, 18309131341807, 18404671443811, 18540013153891, 18559793675383, 18708114170569, 18791804571031, 18819622202461, 18952489549369, 19104713693491, 19185906338047, 19380588947227, 19422676875457, 19456894562011, 19588266676633, 19807154383879, 20141731538941, 20283739587481, 20298888317713, 20328283285939, 20405141488531, 20642469863503, 20848006999843, 21153292318567, 21719845346383, 21846389131351, 21882742319209, 22139028222121, 22835089153267, 23281902018313, 23448129713581, 23912995462123, 24136989378067, 24258011191429, 24606209150533, 25056362656021, 25589395052941, 25604735171689, 25867453860343, 26428280693623, 26606492807497, 26756394917617, 27297134913889, 27872556328987, 28381471439029, 29811331247473, 30042444233173, 30234741319231, 30585529914397, 30711025151179, 32050038769141, 32555079573259, 32701816884031, 33470021902411, 33917681442061, 33942495195607, 34179023285593, 34184127661549, 34898886711517, 36204856496689, 37876637869117, 38145424302301, 39257192244817, 39729605857231, 41060222456347, 42783487794241, 42999011609773, 43234206882379, 44224414680937, 44263178909539, 44827410638851, 45990400423663, 46069343863831, 48712072360423, 48820351674937, 48823378518559, 49389331329199, 49391603993311, 49558389120547, 50342975686651, 54606583422541, 57261577406377, 57579682396777, 58766794301143, 60608646801943, 62729033326591, 63270903057883, 63383334276181, 64067804529319, 67310587507561, 67312991970409, 68234963198341, 70672903493047, 72742294780297, 74759288178217, 74974226441293, 75028242457717, 75781697480263, 77648915837131, 79423227893629, 79884305498611, 80980287295651, 81468825482497, 82280837598697, 82292927039353, 84890215953073, 89248961837743, 90148145162809, 94776140903737, 98603033051827, 109420175594251, 113579640752353, 124044647963101, 126439685146363, 132929049300997, 134430779209141, 137564932144027, 145519550356951, 151619662339111, 154621021097047, 156584637752281, 160076575206721, 162047336419057, 164425816460929, 164453890254637, 180172878410671, 180448762851433, 196681547718307, 230401058568067, 240858850260859, 242404342375171, 242742363565129, 248944267904119, 269856796127407, 294047610769699, 300373428680251, 310133227966381, 312873645397813, 337936995098827, 364303115981989, 371984029318171, 390094486826269, 432641533159903, 450813095260159, 462490133959621, 463449157481137, 507784964149189, 521686527418213, 547862746925617, 555146990584513, 576086723258533, 583527733344427, 649000276552597, 652568485000171, 742561452991081, 743339624461501, 765795792054061, 793857424552669, 802760067710743, 840033765752431, 844473031208419, 874822607465467, 942614634202081, 954412659918733, 968363052131941, 1037457286943029, 1070790660319843, 1133110071744967, 1137204055819063, 1298131759941217, 1309268884232473, 1312155533219491, 1340331056447353, 1412014044618517, 1641984021595243, 1647762521405071, 1670921607814441, 1694765256138931, 1701302270958991, 2077250227840369, 2234520601156213, 2389881331820077, 2407400577709573, 2460477578048341, 2619084740825929, 2817056474149261, 2949176476287967, 3948538479188209, 3961578922097791, 3981315547995397, 4283311287739369, 4608080480597989, 5495782060163023, 5706109080061321, 5797598929133173, 5866243011309991, 6019816946885743, 6189637626631663, 7075809327703903, 7398338934404713, 7570986015124003, 7747981238544499, 7804450894143853, 8196913896774103, 8303312121860731, 8825762386246501, 8910457162652113, 9472183085033743, 10519327229455789, 12001643937692797, 12606215689796749, 13518981554730919, 13648055835205147, 14233476575623573, 14450287680877903, 15169429925253421, 16231502596419529, 17058641515007173, 17161425694503631, 20346640115603833, 20563968995458171, 22170214192500421, 23015628492665857, 24594464180065891, 25138954203734377, 26175692949265549, 28377714852503851, 35035107321996151, 37765606050542101, 47140482657419227, 48700836926686327, 52567682222976871, 57118212413345257, 62489619344090509, 67339766251222879, 69147285938064727, 70029997705437103, 71571862548016297, 82290447059849761, 90999932666010103, 224602598275365931, 254012922572017027, 262969540151048209, 277734552180531169, 320174672811064207, 387759152490278341, 420107179869182083, 478300603363058647, 764059742067641347, 767817669948654781, 942955236066678649, 1565923743128948209, 1980800145615858937, 2762503210701176941, 4798364110889303131, 5036065049491404169}.
